# A dataset-centric review of IoT and IIoT intrusion detection: realism, evaluation biases, and future research directions

**DOI:** 10.3389/fdata.2026.1878260

**Published:** 2026-07-10

**Authors:** Dwarsala Sreedhar Reddy, Kakelli Anil Kumar

**Affiliations:** School of Computer Science and Engineering, Vellore Institute of Technology, Vellore, India

**Keywords:** class imbalance, dataset realism, dataset-centric review, deep learning, Industrial Internet of Things, Internet of Things, intrusion detection systems, machine learning

## Abstract

The rapid growth of IoT and IIoT expands the cyber-attack surface of interconnected and safety-critical systems, and, as such, IDSs have become a fundamental security mechanism. Although very impressive results have been reported for machine learning and deep learning-based IDS in benchmark datasets, these gains often do not generalize to real-world deployments owing to dataset design limitations, realism deficits, and evaluation biases, rather than inherent flaws in detection algorithms, which can lead to significant vulnerabilities in actual operational environments. This study presents a dataset-centric review of widely used intrusion detection datasets from the IIoT, IoT, and traditional network domains. A unified taxonomy differentiates datasets based on the domain context, traffic representation, protocol semantics, and attack modeling assumptions. Based on a common analytical framework, each dataset was reviewed regarding its realism, coverage of the threats, class imbalance, temporal continuity, and modern ML/DL-based evaluation of the IDS. The cross-dataset analysis conducted in this study shows that, in addition to the fact that model architecture and feature engineering play a major role, several studies indicate that the simplicity of the datasets, the class imbalance, and the repetitive attack patterns as well as the evaluation methods can affect accuracy of the IDS. This work further underlines the remaining gaps, such as zero-day and adaptive attacks, limited encrypted traffic, weak temporal evolution, poor support for federated learning, and sparse annotations for explainable IDSs. Finally, this study presents future directions for dataset design aligned with the requirements of next-generation IDSs by highlighting digital twin-based IIoT environments, edge-cloud collaborative data generation, sequential traffic modeling, and explainability-oriented annotations that can ensure robust, trustworthy, and deployment-ready IDS solutions.

## 1 Introduction

### 1.1 Evolution of IoT, IIoT, and cyber-physical systems

The IoT has revolutionized modern computing by allowing the interconnection of heterogeneous devices, including sensors, actuators, smart appliances, and embedded systems, on a large scale. These interconnected environments support the automation of data-driven functions and services across smart homes, healthcare, transportation, and environmental monitoring. Simultaneously, they introduce unprecedented system heterogeneity and scale. With the growth in IoT deployments, the attack surface increases proportionally, with increasing exposure to network-based and protocol-level threats. The IIoT corresponds to a more critical evolution of the IoT, extending connectivity into industrial automation, smart manufacturing, power grids, water treatment plants, and oil and gas infrastructures. IIoT systems are strongly coupled with physical processes and control logics, usually forming Cyber-Physical Systems (CPS), where cyber incidents can directly manipulate physical states to cause safety hazards, equipment damage, or large-scale economic loss. According to recent IIoT-specific dataset development work, domain-sensitive security analysis is required to capture realistic industrial settings and not consumer IoT assumptions (Ferrag et al., 2022). IoT, IIoT, and CPS convergence ensured efficiency of operation and intelligent automation; simultaneously, it increased the risk of insecurity because of constant connectivity, the heterogeneity of devices, and high demands of real-time processing. Such features demand security provisions that are no longer based on the conventional assumption of the enterprise network.

### 1.2 Security challenges in IoT and IIoT environments

The security of IoT and IIoT environments represents a significantly different challenge than the security of conventional IT infrastructures. To begin with, the issue of protocol diversity is among the primary ones since IoT and IIoT systems employ lightweight and domain-specific protocols, for example, MQTT, CoAP, Modbus, and DNP3, the communication semantics of which are not mirrored in existing intrusion detection data sets. The studies of MQTT-based systems also point to the fact that privacy-saving and anonymity communication protocols have a considerable impact on the nature of traffic flows and thus complicate the process of intrusion detection (Shoukat et al., 2025). Resource constraints are also another major contributor. Most IoT/IIoT devices operate with bounded computation, memory, and energy, which limits the application of heavyweight security mechanisms. In IIoT scenarios, security solutions must also be subject to stringent real-time and reliability requirements, where detection latency may disrupt industrial processes. In addition, the ever-increasing usage of encryption and privacy-preserving communication decreases the effectiveness of payload-based intrusion detection. Thus, all modern IDSs increasingly depend on flow-level and behavioral features, shifting the burden of effective detection from purely algorithmic complexity to quality and representative datasets.

### 1.3 Intrusion detection as a dataset-centric problem

Therefore, intrusion detection systems have been at the core of most IoT and IIoT security architectures because of their capability to detect unknown and evolving threats. Recent research has mostly adopted machine learning and deep learning techniques, often reporting very high accuracy in detection. However, analytics performed using various popular IDS benchmarks are often driven by the properties of the datasets rather than the true detection capabilities. For instance, critical analyses of various IoT botnet datasets, which are in wide use today, indicate that class imbalance, repetitive attack patterns, and feature redundancy can result in the inflation of accuracy together with poor generalization. Both studies show that various IDS models learn dataset-specific artifacts rather than meaningful malicious behaviors, which raises concerns about real-world applications (Peterson et al., 2021). In another study by feature engineering and the selection, it is further noted that the performance improvement is closely tied with the dataset structure as opposed to model intelligence (Fernando et al., 2024). Classical benchmark datasets, including NSL-KDD and CIC-IDS2017, remain very popular although they were designed on legacy enterprise networks. Studies of federated-learning-based IDS have also established that the modern learning paradigms despite their promise are still constrained by old-fashioned, non-IoT datasets that restrain their applicability to IoT and IIoT contexts (Amin et al., 2025). Therefore, IDS challenges are becoming more data-oriented, as opposed to model-oriented.

### 1.4 Limitations of the existing dataset-oriented surveys

The state-of-the-art IDS surveys are largely grounded on either enumerating different datasets or synthesizing the reported performance measures. They usually would like to use IoT, IIoT, and traditional network datasets interchangeably. The practice blurs the basic distinctions between protocol semantics, traffic behavior, and operational constraints across domains. Analytical analyses of major datasets warn against blind benchmarking on highly imbalanced datasets which may be misleading to infer about the effectiveness of the IDS (Fukushima et al., 2025). More so, lack of dataset-model compatibility is rarely considered in most studies where the datasets are processed in the complex DL architecture without the necessary temporal consistency and local contextual nuances. Recent research on explainable IDS emphasizes that the richness of a dataset has a direct impact on the interpretability and the level of trust, and the practical use of contact traces is limited by the absence of annotations to facilitate explainable decision-making (Hassini et al., 2024). On the other hand, methods of dealing with imbalance, like oversampling, can create performance numbers artificially high but not correspondingly increased in the sense of detection strength, exacerbating the results further (Kalifati et al., 2025). These limitations make a critical dataset-centric review necessary-one that evaluates not only what datasets exist but also how their design choices shape IDS research conclusions.

### 1.5 Contributions and novelty of this review

These challenges are the reasons why this review carries out a systematic and critical review of intrusion detection datasets registered within IoT, IIoT, and IDS spheres. This study emphasizes dataset realism, threat representation, protocol semantics, and evaluation bias as key determinants of IDS effectiveness, unlike previous surveys. The key contributions of this study are as follows:

- A structured taxonomy of IoT and IIoT intrusion detection datasets with respect to the domain context, traffic representation, protocol depth, and threat realism.
- A critical analysis shows that high IDS accuracy often reflects the imbalance and simplicity of the dataset rather than genuine security capability.
- Examining dataset–model mismatch: a study of the inappropriate use of ML/DL architectures on unsuitable datasets.
- Identification of open research gaps and future dataset requirements, including encryption-aware detection, explainability, federated learning, and temporal evolution.

Table 1 shows that this review differs from earlier IDS surveys, which mostly focus on summarizing the datasets and the performance of the models, by taking a dataset-centered approach that examines the realism of the datasets, temporal continuity, protocol semantics, encrypted traffic representation, explainability support, federated learning readiness, and evaluation bias. In addition, a quantitative dataset realism scoring framework is presented which can help to objectively compare benchmark datasets.

**Table 1:** Comparison of existing IoT/IIoT IDS surveys with the present review.

Feature	Existing surveys	This review
IoT dataset survey	✓	✓
IIoT dataset survey	Partial	✓
Evaluation bias analysis	✗	✓
Dataset realism assessment	✗	✓
Temporal continuity analysis	✗	✓
Encrypted traffic analysis	✗	✓
Explainability assessment	Partial	✓
Federated learning assessment	Partial	✓

The review focuses on the realism of the dataset rather than benchmarking centered on algorithms and calls for the development of more reliable and deployable IDS solutions for next-generation IoT/IIoT systems.

## 2 Background and taxonomy

### 2.1 The Internet of Things (IoT), the Industrial Internet of Things (IIoT), and Intrusion Detection Systems (IDS)

**Figure 1:**
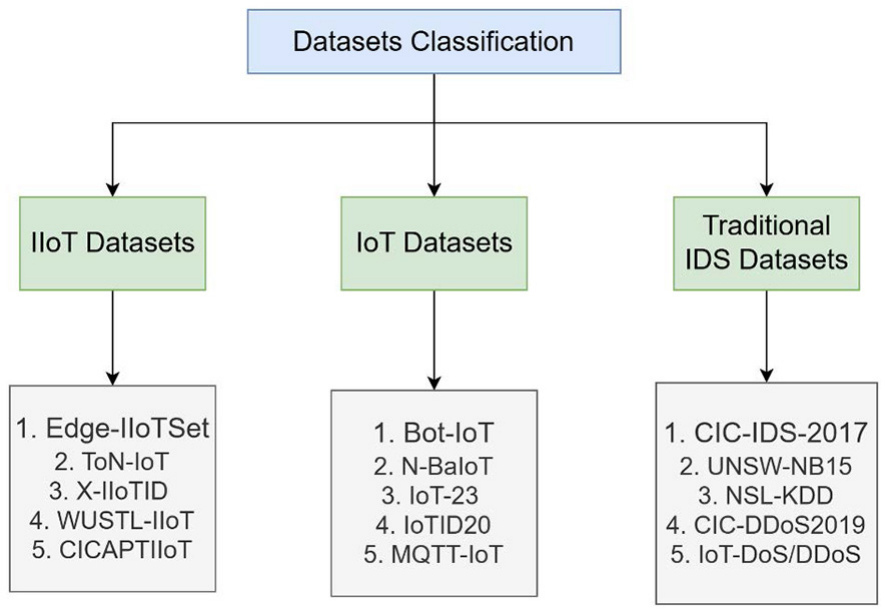
Domain-based classification of IoT, IIoT, and traditional IDS datasets.

Figure 1 illustrates the domain-based Classification of IoT, IIoT, and Traditional IDS Datasets. IoTs are large-scale networks of interconnected physical objects, such as sensors, actuators, and smart devices, that communicate over the Internet to collect, exchange, and process data. IoT systems are widely deployed in consumer and service-oriented domains, such as smart homes, healthcare monitoring, agriculture, and intelligent transportation. These environments are characterized by high device heterogeneity, lightweight communication protocols, and frequent device churn.

The IIoT is a niche and critical evolution of the IoT, in which connected devices are deployed in industrial and operational environments, such as smart manufacturing, power grids, water treatment plants, oil and gas infrastructure, and industrial automation systems. Unlike consumer IoT systems, IIoT systems are tightly associated with physical processes and industrial control logic. Even minor cyber incidents in IIoT environments can result in production downtime, equipment damage, safety hazards, or large-scale economic losses. Thus, the IIoT security requirements are far greater than those of general IoT systems. Intrusion Detection Systems (IDSs) form part of the fundamental security aspects of both IoT and IIoT systems, as they utilize them to identify the behavior of the network or system to identify suspicious activities. Nevertheless, IDS solutions design and evaluation are closely related to the area they are used. Many IDS created to be used in enterprise IT networks cannot be applicable in the IoT and IIoT context (Table 2) due to variations in protocols, timing, and traffic patterns, as well as resource availability. The differences in these domains have impact on the dataset design. IoT data are usually representative of network traffic diversity and device-level activity, and IIoT data needs to indicate the protocol semantics, deterministic communication patterns, and associations between cyber events and physical phenomena. Using IoT, IIoT, and traditional IDS datasets interchangeably will result in false evaluation results and unrealistic performance claims.

**Table 2:** Comparison of IoT, IIoT, and IDS Contexts.

Aspect	IoT	IIoT	IDS implications
Application domain	Consumer and services	Industrial and critical infrastructure	IDS must be domain-aware
Protocols	MQTT, CoAP, and HTTP	Modbus, DNP3, OPC-UA, MQTT	Dataset protocol coverage matters
Timing constraints	Flexible	Strict real-time	Temporal realism is critical
Security impact	Privacy, service disruption	Safety, physical damage	Evaluation must reflect severity
Dataset design focus	Network behavior	Process-aware traffic	IDS datasets must differ

### 2.2 Intrusion detection system paradigms and dataset dependency

**Figure 2:**
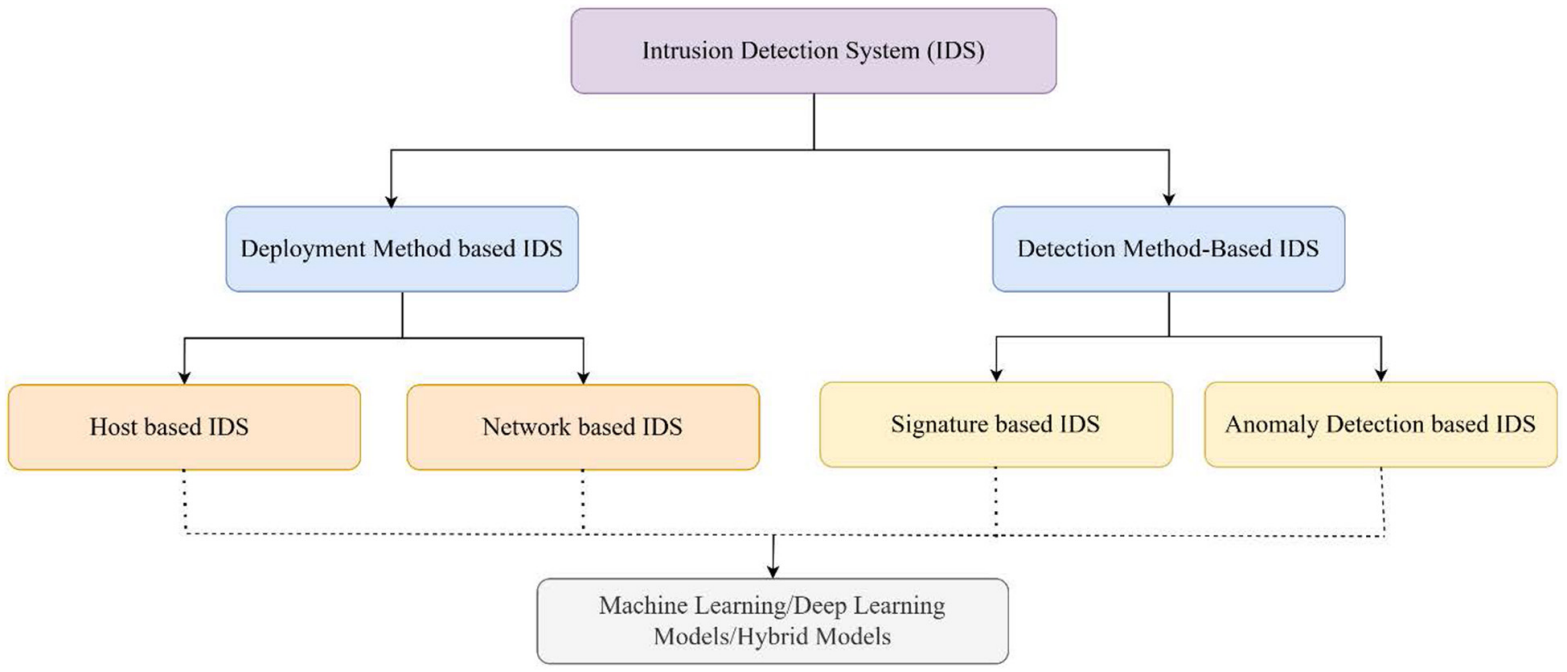
Top-level taxonomy of intrusion detection systems paradigms, and their combination with machine learning, deep learning, and hybrid detection models.

IDSs are generally classified into signature-based, anomaly based, and hybrid approaches. Each paradigm imposes fundamentally different demands on dataset construction and labeling, making the dataset characteristics a decisive factor in the effectiveness of IDSs. Figure 2 shows the structured taxonomy of intrusion detection systems. IDS approaches are categorized based on two fundamental dimensions: deployment method and detection strategy. Deployment-based IDS defines where the data are collected. Host-based IDS record system-level activities, such as logs and process behavior, and then analyze them, whereas network-based IDS monitor packet-level or flow-level network traffic. Detection-based IDS defines how intrusions are identified. Signature-based approaches utilize known attack patterns, whereas anomaly-based approaches model deviations from normal behavior.

This representation shows that the performance of ML/DL-based IDSs is inherently limited by the data collected and the detection logic determined by the dataset design. Thus, the taxonomy strengthens the key argument of this review: dataset characteristics defined by approaches of IDS deployment and detection determine the validity, generalization capability, and applications in real-world settings of an IDS, with a prime focus on IoT and IIoT scenarios. Signature-based IDS depend on predefined attack patterns or rules to identify malicious activities. Therefore, datasets for evaluating signature-based IDS must provide accurate and exhaustive labels for attacks. Such systems are thus effective only against already known threats; they cannot detect 0-day attacks or new intrusion strategies by design, since their detection capability is bounded by prior knowledge encoded in signatures. In general, anomaly-based IDS attempt to learn normal system behavior and detect deviations that may indicate intrusions. This paradigm places much stronger demands on datasets, especially when it comes to benign traffic realism. A dataset used for IDS must contain normal behavior over a long duration and be representative, reflecting the actual operational conditions. Poorly designed datasets with biased or constrained normal traffic are likely to result in high false-alarm rates, especially in dynamic IoT and IIoT systems. To establish a middle ground between detection accuracy and adaptability, hybrid IDSs are composite signature-based and anomaly-based. Nevertheless, they will be successful only with datasets that entail real-world benign behavior and various well-labeled attack scenarios. Datasets that are missing one of the two components may provide biased estimates that favor one of the detection mechanisms and conceal the flaws in the other. These observations suggest that it is impossible to study IDS paradigms outside the context of datasets. A dataset that can be used in one IDS paradigm may necessarily be inadequate in another, which makes data-conscious studies of IDS a necessity.

### 2.3 Machine learning and deep learning in IDS: dataset-centric perspective

Recent studies have been overtaken by ML and DL technologies because they are the most suitable for current IDS due to their internal ability to represent complex trends in network traffic. Traditional ML algorithms, such as Random Forest, Support Vector Machine, and gradient boosting methods, will remain widely used because of their effectiveness in structured, tabular feature spaces commonly derived from flow-based datasets.

**Figure 3:**
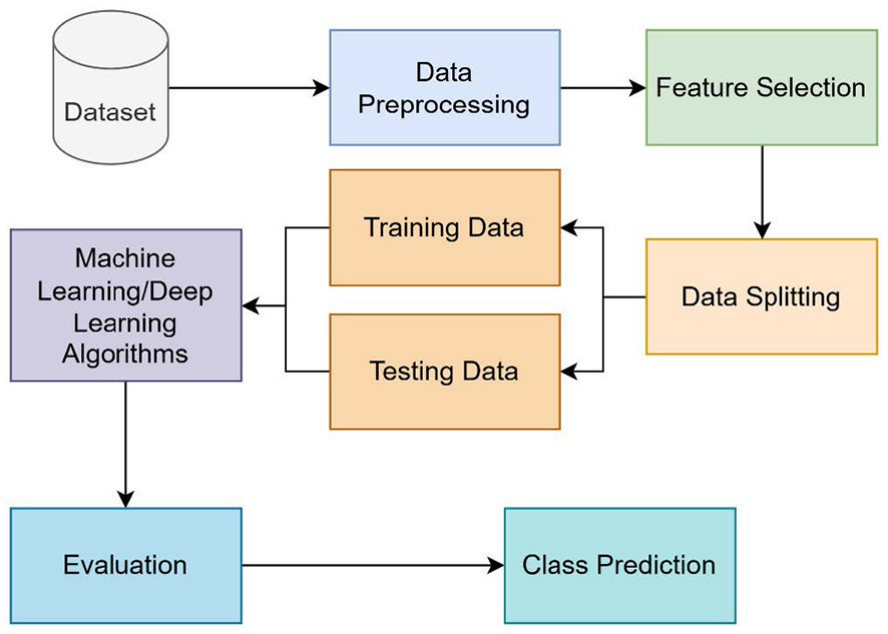
Dataset-centric view of machine learning and deep learning-based intrusion detection systems.

The dataset-centric workflow of ML/DL-based IDS is shown in Figure 3 and it explains how the IDS performance and generalization ability are affected by the four factors related to dataset: distribution, temporal structure, feature selection, and preprocessing. Consequently, complex architectures tend to learn dataset-specific idiosyncrasies rather than true intrusion patterns, leading to deceptively high accuracy but poor real-world generalization. Deep learning methods, such as CNNs, RNNs, LSTMs, and Transformer models, are increasingly used to model the spatial and temporal dependencies of traffic data. However, these models are often presented as being much better than they are because the benefits that can be obtained with a given dataset are limited. Most existing IDS datasets only include flow-level features, which are aggregated and lack rich temporal continuity, rendering them unsuitable for sequence models such as LSTMs and Transformers.

In such cases, complex DL architectures learn dataset-specific artifacts rather than intrinsic patterns of intrusion. Moreover, extreme class disparity, predominant in IoT and IIoT datasets, may artificially amplify accuracy values and is a characteristic that covers poor performance within the minority classes of attack. Specialized transformer-based models require rich contextual information and long-range sequential data to successfully utilize self-attention mechanisms. Such long-horizon dependencies are not maintained by most available IDS datasets, which poses several methodological validity issues when using attention-based models in such situations. The quality of ML/DL-based IDS is bound to the quality and representativeness of the datasets on which the system operates. The fact that this observation also underscores the thesis of this review, namely that the quality, realism, and structure of the dataset are frequently more powerful than the complexity of the model in generating performance in ID.

### 2.4 Taxonomy rationale for IDS dataset analysis

Table 3 summarizes the key dimensions used in the proposed dataset taxonomy, along with an explanation of how each dimension influences the IDS evaluation and model suitability. This review addresses inconsistencies in IDS evaluation by adopting a dataset taxonomy that explicitly links the application domain, traffic representation, protocol specificity, and learning paradigm suitability. Grounding ML/DL usage in dataset characteristics, taxonomy prevents cross-domain misuse and enables fairer comparison across IDS studies.

**Table 3:** Taxonomy dimensions and their implications for IDS evaluation.

Taxonomy dimension	Description	IDS evaluation implication
Application domain	IoT, IIoT, or IT context	Prevents cross-domain dataset misuse
Traffic representation	Packet-level or flow-level	Determines feasibility of DL sequence models
Protocol specificity	Generic vs. domain protocols	Ensures semantic realism
Temporal structure	Static vs. sequential	Affects validity of LSTM/Transformer
Learning suitability	ML vs. DL readiness	Enables fair model comparison

## 3 Related work

IDS techniques have considerably evolved for IoT and IIoT environments, mainly driven by the emerging sophistication of cyber threats, high traffic volume, and resource constraints implicit in IoT devices. The current state of the art can be broadly summarized in the following sections: classical machine learning-based IDS, deep learning-based IDS, hybrid and ensemble approaches, federated and online learning-based IDS, explainable IDS, and dataset-centric and survey studies.

### 3.1 Machine learning-based IDS

Hasan et al. (2025) demonstrated that using XGBoost and MLP models, a high accuracy of over 98% in classification with five selectively chosen features of CIC-DDoS2019 was possible. Although their methodology lowers the computational cost and enables lightweight IoT deployments, their main priority is the reduction of features rather than the reduction of dataset bias or adversarial resistance. Gebrye et al. (2025) proposed feature extraction and Boruta-based selection methods to detect multi-class flooding DDoS in IoT networks with over 90% accuracy on recent datasets. The current study focuses on balanced assessment and strong analysis. Vibhute et al. (2024b) studied the problem of network anomaly detection with the NSL-KDD benchmark dataset and suggested a feature selection mechanism based on a Random Forest with further classical machine learning classifiers (SVM, Logistic Regression, and KNN). Their method obtained the highest possible validation of 98.24 when using the KNN. Thana-Aksaneekorn et al. (2024) applied feature reduction and XGBoost-based classification on NSL-KDD, where 99.72% accuracy was achieved with imbalance correction using SMOTE. Although, this study has shown excellent results in terms of ensemble learning and oversampling. (Gheni and Al-Yaseen 2024) proposed a two-step clustering-based IDS with CICIoT2023 data and GSK optimization for dimensionality reduction. The framework was better than previously proposed frameworks, as it enhanced feature selection and detection performance. Elmahfoud et al. (2024) performed a comparative analysis of classical machine learning algorithms (DT, RF, KNN, AdaBoost, and SVM) to identify intrusion in the IoT on the IootID20 dataset. Their findings showed that the highest accuracy (99.80%) was obtained using the decision tree classifier. Although the study provides some important benchmarking insights, real-time deployment challenges, the imbalance of data, and adaptive security mechanisms are not discussed in dynamic IoT conditions. Kaushik et al. (2025) suggested a lightweight IDS model in which statistical feature selection methods are used to minimize the cost of computation. Although efficient, the main focus of the study was feature optimization without any adaptive or federated learning strategy to deal with distributed IoT. Saied et al. (2023) conducted a comparative study of ensemble tree-based algorithms to detect IoT botnets. Their results indicated that the multi-class classification accuracy of Random Forest was close to the required perfection (0.999991). This study emphasized that machine learning has computational benefits for deep learning in restricted IoT systems. Nevertheless, the study was restricted to tree-based ensemble techniques and did not consider deep representation learning and cross-layer IoT security modeling.

### 3.2 Deep learning-based IDS

Akgun et al. (2022) presented a DDoS intrusion detection model based on deep learning and tested it on the CIC-DDoS2019 dataset. The focus of their work was on preprocessing methods, such as feature elimination, duplicate removal, and normalization, and they obtained a binary accuracy of 99.99%. Nevertheless, the analysis was based on one dataset and was not conducted on cross-dataset generalization and unknown attack cases. Bandarupalli (2025) proposed a lightweight CIC-IDS2017-based DNN-based anomaly detection model with a focus on computational efficiency but low detection rates. Xu et al. (2021) presented an autoencoder-based anomaly detection model with five layers and improved preprocessing and reconstruction loss optimization. They achieved better detection accuracy (90.61) and F1-score (92.26) on the NSL-KDD dataset. Luqman et al. (2025) offered a smart in-network IDS on IoT based on UNSW-NB15 and BoT-IoT data using Random Forest, SVM, and LSTM algorithms. Through the combination of datasets and feature engineering, they achieved a maximum of 99.97 multi-class accuracy. Inuwa and Das (2026) innovated a better Fully Connected Neural Network (FCNN) architecture with residual connections, attention, and SHAP-based interpretability for detecting anomalies in IoT. The model was almost perfect for the ToN-IoT and UNSW-NB15 datasets. One of the strengths is that AI integration can be explained; however, the computational performance and ability to deploy edges on edges need to be validated further. Narayan et al. (2024) addressed minority-class bias with a hybrid CNN-BiLSTM IDS model with SMOTE-Tomek balancing on NSL-KDD and UNSW-NB15. This strategy substantially enhanced the F1-scores of the minority classes. Although class balancing was found to be fairer, deep hybrid models are more complex to train and are not necessarily suitable for lightweight impairments of the Internet of Things (Vibhute et al., 2024a) tested CNN based anomaly detection on UNSW-NB15 with Random Forest feature reduction with a testing accuracy of 99.00%. Although the study established that CNN was effective in network intrusion detection, it failed to address class imbalance and IoT-specific traffic heterogeneity. Hosseini et al. (2025) proposed CNN-CBAM-GRU variants of intrusion detection over UNSW-NB15 and NSL-KDD datasets, which perform better in multi-class classification (99.56%). Aishwarya et al. (2025) presented a proposal of an integrated AI-based Internet of Vehicles (IoV) generative IDS, which uses LLM frameworks to handle heterogeneous vehicle communication data. The model demonstrated a high score in the TONIoT and CICIoV2024 datasets. However, the methodology is domain-specific to car settings and might be problematic in general IoT systems with low scalability. Khan and Mian (2025) presented a self-supervised low-latency masked autoencoder (MAE) intrusion detector based on entropy-based masking and LightGBM on IIoT systems. The model solves the problems of class imbalance and label scarcity and has a much lower inference time than state-of-the-art solutions. Diaba et al. (2023) introduced GSFTNN, a Genetically Seeded Flora Transformer Neural Network to detect SCADA intrusion by the WUSTL-IIOT-2018 dataset. However, its high model complexity and transformer architecture can cause constraints in the implementation of IIoT operations in latency-sensitive control loops. Altunay and Albayrak (2023) presented a hybrid CNN+LSTM intrusion detection model for IIoT networks using UNSW-NB15 and X-IIoTID. Their hybrid architecture showed better binary and multi-class classification performance compared to standalone CNN or LSTM models (Almalki et al., 2025). BoT-IoT trained 2D CNN models based on the BoT-IoT 2020 dataset using tabular IoT intrusion data. By reorganizing the tabular features into grid formats and optimizing the convolutional layers, a detection accuracy of approximately 99% was attained. Fares et al. (2025) presented TFKAN, a Transformer-based model that replaces MLP layers with Kolmogorov Arnold Networks (KANs) to design an IoT IDS. The proposed model demonstrated a maximum accuracy of 99.96 on RT-IoT2022 with a decrease in the number of parameters by 78%. Abbasi et al. (2025) presented a dimensionality reduction process of an Internet of Things botnet detection model based on the Fisher score of feature selection, autoencoders, and deep neural network models. They used this approach, and their version, tested on the N-BaIoT dataset, obtained a maximum accuracy of 99 percent in binary and multi-class classification. Although the study showed the efficacy of feature ranking in device-specific intrusion detection, it was based on per-device optimization, and it did not undergo cross-dataset validation. Sharma et al. (2025) presented a deep neural network self-attention-based system augmented with a learnable feature gating system to obtain interpretable IoT intrusion detection results. Their model was evaluated on the BoT-IoT and N-BaIoT datasets and showed accuracies of 99.3% and 99.6%, respectively, and used SHAP and LIME to interpret its results. Although the current study contributes significantly to the interpretability of IoT IDS, it uses centralized deep learning architectures and does not consider the diversity of ensembles and hybrid modeling approaches. The ASEADOS-SDN-IoT dataset, which is a hybrid dataset, was created by (Yasarathna and Le-Khac 2026) by integrating OpenFlow-based SDN control with IoT device traffic to simulate realistic scenarios for benchmarking ML- and DL-based IDS systems. The dataset has 457k labeled flow data with 83 features, covering various types of attacks.

### 3.3 Hybrid and ensemble IDS approaches

Nguyen and Le (2023) presented a hybrid supervised-un-supervised system that integrated SOCNN, LOF, and iNNE as one unit to identify unknown DoS/DDoS attacks. Their strategy showed high resilience to adversarial attacks, as their F1-scores were high in benchmark datasets. The current study recognizes these limitations of datasets and focuses on the rigor of validation. Kayyidavazhiyil (2023) presented a more improved approach of Genetic Sine Swarm (E-GSS) feature selection strategy with a Deep Meta-Heuristic Artificial Neural Network (DMH-ANN) classifier to intrusion detection on UNSW-NB15 and NSL-KDD datasets. The model used dealt with both the optimization of features and the accuracy of detection. Lazzarini et al. (2023) proposed DIS-IoT, an ensemble of stacking deep learning models to intrusion detection on ToNIoT, CICIDS2017, and SWaT datasets. Their method showed a higher classification accuracy in multi-class classification using deep ensemble stacking (Asif, 2025). UNSW-NB15 introduced OSEN-IoT, a streamlined stacked ensemble network that integrated DenseNet121, MobileNetV2, and ResNet50V2 and used genetic algorithm optimization in heterogeneous IoT threat detection. The model had high detection accuracy on the Edge-IIoTset and UNSW-NB15 datasets. The performance benefits are substantial, but edge deployment may be restricted because of the heavy architecture, and it is not easily interpretable. Vinta et al. (2025) proposed QBCMVT, a Quantum-based Coati-MobileViT IIoT intrusion detection network. It uses the Quantum-based Coati Optimization Algorithm (QCOA) to select the features and a lightweight MobileViT deep learning classifier and combats the issue of class imbalance through TGAN-based augmentation on the Edge-IIoT and WUSTL-IIoT-2021 datasets. Ye et al. (2024) offered an ensemble model that used Cooperative Co-evolution Improved Hybrid Breeding Optimization (CCIHBO) to select features in IDS applications. The method was tested on the NSL-KDD, WUSTL-IIoT, and HAI datasets, and it improved the detection rate and alleviated the curse of dimensionality. Mir and Trik (2025) proposed a hybrid GCNGRU intrusion detection framework that was optimized using Ant Colony Optimization (ACO). The framework works well for capturing the structural dependencies of with GCN and time trends of with GRU on the Edge-IIoTset and WUSTL-IIoT datasets. Sinijoy et al. (2026) described a digital-twin-based SCADA and IIoT multilevel-based attack detection and prevention system. In the proposed solution, anomalies in a digital twin environment are identified by CNN-NGBoost-NaïveBayes classifiers that have been optimized with Sandpiper Optimization and GAN-ANN models. By employing a multidomain learning system, Babayigit and Abubaker (2024) addressed the issue of cross-dataset generalization and proposed a learning system using the Edge-IIoTSet, WUSTL-IIoT-2021, and X-IIoTID datasets. The study is useful in enhancing transferability in varying IIoT scenarios through autoencoder-based dimensional matching and CNNGRU hybrid categorization involving Bayesian hyperparameter optimization. Al-Shurbaji et al. (2025) developed BoT-EnsIDS that optimally employs bio-inspired feature selection (PSO and GTO), GAN-driven data augmentation, and a hybrid CNN-LSTM classifier to detect IoT botnets using the BoT-IoT dataset Ensemble optimization enhanced the relevance of features and their detection (≈97% accuracy). However, the high optimization cost increases the complexity of the computations, which may limit the deployment of edges in real time. Qaddos et al. (2024) proposed a hybrid CNNGRU intrusion detection system that is specialized in and IoT security. The authors successfully removed the issues of class imbalance with the help of feature-weighted SMOTE (FW-SMOTE), which resulted in accuracies of 99.60 and 99.16 on IoTID20 and UNSW-NB15, respectively. Bibers and Abdallah (2025) presented a powerful ensemble learning system that combines bagging, boosting, blending, and stacking anomaly detection in IoT systems. Their comparison of the MEMS and N-BaIoT sets proved that the ensemble models were much better than the single classifiers, with a mean accuracy of 95.53 on N-BaIoT. Although their framework enhanced robustness and comparative benchmarking among models, it was not based on attention-based learning of features and explanatory AI elements.

### 3.4 Federated and online learning-based IDS

Anley et al. (2025) proposed FELACS, a federated architecture of learning with adaptive customer selection, to detect IoT DDoS. Their solution enhances the rate of convergence and privacy protection in heterogeneous IoT environments. Zhou et al. (2026) presented a federated transfer learning architecture (FtKD) built upon two-stage knowledge distillation to detect IIoT intrusion. The method tackled non-IID data issues and privacy protection with the help of entropy-based aggregation and differential privacy and demonstrated high results on ToN-IoT and Edge-IIoTset data. Anwer et al. (2025) proposed a federated learning-based CNN-BiLSTM architecture to overcome scalability and privacy challenges. Their model was tested on the X-IIoTID, WUSTL-IIoT, and Edge-IIoTset datasets, and with a high detection accuracy, they used less communication overhead. The current study builds on this line and includes explainability and greater architectural adaptability in a single IoT security framework. Xie et al. (2025) presented DTKD-IDS, a two-teacher knowledge distillation framework that minimizes model complexity in IIoT intrusion detection. Using prototype and complementary distillation mechanisms, the student model considerably reduced the parameters and achieved high detection rates in both the X-IIoTID and NSL-KDD datasets. The study lacks both explainability and cross-dataset manifold alignment strategies, which would have enhanced its effectiveness. Zou et al. (2025) presented a knowledge distillation framework with edges to assist a federation (E-FPKD) to prevent cyber-attacks in an EV charging station based on prosumers. They combined federated learning, knowledge distillation, and prototype aggregation to mitigate non-IID data and privacy issues and outperformed the Overall Detection Correctness (ODC) on the NSL-KDD, UNSW-NB15, and IoTID20 datasets.

### 3.5 Explainable and graph-based IDS

Jia et al. (2025) IDEAL-an explanation-guided learning malicious traffic-detection model proposed that combines domain knowledge in model training. The study enhanced performance and interpretability by using Snort rule-based automatic annotation and loss of tasks with explanation supervision. Saheed and Misra (2025) introduced CPS-IoT-PPDNN, an explainable and privacy-respectful DNN architecture over CPS-enabled IoT networks with SHAP-based explanations. Their model showed almost perfect results on the Edge-IIoTset and X-IIoTID datasets, with a focus on resilience and differential privacy. Alani (2023) created an explainable and efficient flow-based IIoT IDS, E2I3DS, which can be used to reduce the features of WUSTL-IIoT-2021 by 48, yet achieving 99.97% accuracy. Although the framework is efficient and interpretable, it does not apply to the imbalance of datasets or cross-domain generalization between heterogeneous IIoT datasets.

Sudhakar et al. (2025) proposed a Dynamic Diffusion Spatial-Temporal Graph Convolutional Network (DDSGCN) optimized through the Starfish Optimization Algorithm (SFOA) to discover anomalies and self-heal IIoT systems. The framework helps improve the accuracy of anomaly detection and fault recovery time by utilizing graph-based spatiotemporal modeling on WUSTL-IIoT-2021. Sadhwani et al. (2025) suggested a multi-class explainable IDS model for IoT settings based on CNN, LSTM, and Bi-LSTM architectures and SHAP-enhanced feature interpretation. The experiment showed better model transparency and a lower feature subset without a major loss of accuracy in the NSL-KDD, TON-IoT, UNSW-NB15, and X-IIoTID datasets. The current study builds upon this by incorporating multi-domain learning processes and explainable modeling.

### 3.6 Dataset-centric and survey studies

Hozouri et al. (2025) performed a thorough review of IDS that utilize ML and DL methods using datasets such as CIC-IDS2017 and UNSW-NB15. Despite the general description of the study on the architectures of the IDS and learning models, no critical analysis of inconsistencies in the datasets and the lack of validation is provided. Liu et al. (2022) critically analyzed the errors and inconsistencies in the CIC-IDS2017 and CSE-CIC-IDS2018 datasets. Their results showed undocumented problems with the orchestration of attacks, creation of features, and labeling, which questioned the validity of many published results. The current research extends this fact by focusing on the prudent selection of datasets and transparency in validation in the study of IoT intrusion detection. Alatram et al. (2023) presented the DoS/DDoS-MQTT-IoT dataset, which was specifically created to test MQTT-based IoT traffic. The current study is not the end of protocol-specific analysis but a progression to an even more detailed threat model. Kadri et al. (2024) presented a survey and taxonomy of DoS/DDoS detection methods in IoT systems, surveying 80 articles and categorizing validation methods, data, and metrics. The current study adds to this taxonomy by including an empirical robustness assessment. Shafi and Lashkari (2025) have created BCCC-IoT-IDS-Zwave-2025, which is a large-scale smart home dataset designed to address key limitations of existing IoT datasets by incorporating diverse protocols, device types, and over 80 types of attacks. The dataset contains various types of telemetry data, including IP traffic, Z-Wave communication, MQTT, and device activity, which can be used to benchmark next-generation IoT-based IDS systems.

Moustafa (2021) released a distributed testbed framework to assess AI security systems at the edge and suggested the TONIoT dataset. To provide a solution to the shortage of heterogeneous and realistic IoT datasets, this study combined SDN, NFV, and service orchestration to create a simulation of real-world attack scenarios. This study follows this line of thought by highlighting adaptive learning processes and explainability in heterogeneous IoT systems. Dhirar and Hamad (2025) performed a comparative analysis of a new SDN-IoT IDS dataset with BoT-IoT, ToN-IoT, and InSDN using CNN, LSTM, RNN, and DNN models. They focused on dataset realism and model performance. Although it is useful for performance comparison, the study is mostly aimed at dataset analysis instead of architectural innovation or deployment optimization. Zeeshan et al. (2022) presented a Protocol-Based Deep Intrusion Detection (PB-DID) model against DoS and DDoS attacks based on UNSW-NB15 and BoT-IoT data. Their method addressed the problem of imbalance and achieved 96.3% using deep learning. However, it is still only protocol-specific attack detection with no multi-class multi-threat modeling of IoT. The message security (communication security) side says, Saqib and Moon (2024) introduced a multi-factor authentication code for lightweight MQTT-based IoT applications using elliptic curve cryptography (ECC) and fuzzy extractors. However, this study lacks intrusion detection and anomaly analysis mechanisms and mainly deals with authentication. Kaganurmath and Cholli (2025) presented a mechanism called Dynamic Lightweight Authentication for MQTT (DLA-MQTT), which relies on the use of a GFSR-based pseudo-random number generator to generate ephemeral keys. Their solution enhances resource use and reduces frequent attacks, such as MitM and DoS. However, it emphasizes essential management and secure communication instead of comprehensive intrusion detection or preventive dynamic learning. Saha et al. (2024) created a performance analysis model of MQTT client libraries, focusing on the latency, scale, and use of resources over IoT-based manufacturing applications. Although the framework provides an understanding of the efficiency of MQTT communication, it does not discuss security vulnerabilities or intelligent threat detection systems.

## 4 Research methodology

### 4.1 Research design and review philosophy

This paper takes a systematic dataset-centric review approach to critically analyze intrusion detection datasets that are used in Internet of Things (IoT), Industrial Internet of Things (IIoT), and Cyber-Physical System (CPS) security research. Unlike the traditional survey study which primarily summarizes benchmark data and reported detection rates, the central determinants of reliable intrusion detection research in this review are analytical synthesis, dataset realism, evaluation reliability and methodological validity. The methodological design of this review is informed by the fact that the capabilities of the databases, and not just the complexity of the algorithms, more restrict the effectiveness of modern machine learning (ML) and deep learning (DL)-based intrusion detection systems (IDSs). This paper therefore analyzes the impacts of dataset construction strategies, protocol semantics, traffic representation, temporal continuity, class imbalance and orchestration of attacks on the effectiveness and real-world applications of IDS frameworks reported. The review is informed by the Preferred Reporting Items of Systematic Reviews and Meta-Analyses (PRISMA) guidelines on article identification, screening, eligibility assessment and final inclusion. The overall methodology workflow includes a systematic literature search, a systematic data extraction, taxonomy building, a comparative analytical synthesis, a bias quantification, and a cross-dataset analysis. The methodology was developed in such a manner that the next objectives would be attained:

- To identify and categorize in a systematic manner popular intrusion detection datasets of the IoT and IIoT.
- To create one taxonomy between the features of datasets and the paradigms of the assessment of paradigms of the IDS.
- To critically analyze limitations of realism and evaluation bias issues, on benchmarking practices of IDS, the following questions will be formulated:
- To explore the correlation between characteristics of datasets and performance of IDS as it has been reported.
- To realize the future data set requirements to realize reliable, scalable, explainable, and deployment ready IoT/IIoT IDS research.

### 4.2 Research questions

The review was conducted with a set of research questions which were aimed at exploring the correlation between dataset design and the reliability of IDS evaluation.

These research questions in Table 4, become the basis of analytical framework of the proposed dataset-based evaluation framework and guide the comparative analysis carried out within the framework of the current study.

**Table 4:** Research questions and objectives of the IoT and IIoT intrusion detection research systematic dataset-centric review data.

Research question	Objective
RQ1	What are the most common datasets in the existing studies concerning IoT and IIoT intrusion detection?
RQ2	What are the impacts of dataset characteristics over the effectiveness appraisal of ML/DL-based IDS?
RQ3	What constraints of realism and bias in evaluation do modern IoT/IIoT IDS datasets have?
RQ4	What are the dataset features of stable and reliable IDS benchmarking?
RQ5	What are the future research directions of next-generation IoT and IIoT intrusion detection datasets?

### 4.3 Systematic literature acquisition strategy

The acquisition of literature was systematic and wide-ranged to cover the recent advancements in IoT, IIoT, CPS, and intrusion detection research. Peer-reviewed journal articles, conference papers, and quality benchmark studies published between 2016 and 2026 were restricted to the search. The literature search has been performed in the following scientific databases:

- Scopus
- Web of Science
- IEEE Xplore
- ScienceDirect
- SpringerLink
- ACM Digital Library

To obtain the maximum coverage of retrieval and maintain topic specificity, Boolean combinations of keyword queries were built around the following words: IoT intrusion detection, IIoT cybersecurity, dataset benchmarking, machine learning-based IDS, federated intrusion detection, explainable security analytics. The search query was created to find relevant research articles on IoT, IIoT, intrusion detection systems (IDS), datasets, and the latest AI-based security solutions.

Purpose of the search query:

- To identify high-quality research papers related to IoT and IIoT intrusion detection.
- To collect studies involving IDS datasets and cybersecurity evaluation.
- To include machine learning and deep learning-based IDS approaches.

### 4.4 Study selection criteria

To ensure that methodological uniformity and analytical applicability, we had worked on the inclusion and exclusion criteria prior to the screening process.

#### 4.4.1 Inclusion criteria

To be the subject of the studies, it is needed to fit one or more of the following requirements:

- Published between 2016 and 2026.
- Journal article or conference publication that has been peer reviewed.
- Data used is IoT, IIoT, CPS, or IDS.
- Included experimental intrusion detection evaluation.
- Statistical reports of datasets, attack modeling methodologies, or the methods of benchmarking IDS.

Researched on machine learning, deep learning, or federated learning, or explainable IDS paradigms.

#### 4.4.2 Exclusion criteria

Studies that were excluded based on the following criteria:

- Recent publications on some databases.
- Non-English publications.
- Posters, theses, non-peer reviewed reports like tutorials, editorials.
- Cryptographic or authentication studies which are unrelated to IDS.
- Research without adequate experiment or data.
- Conventional enterprise IDS investigations are not IoT or IIoT applicable.

These criteria were to contain only methodologically relevant and experimentally validated studies in analytical synthesis.

### 4.5 Screening and eligibility assessment

The article screening and eligibility assessment process based on PRISMA followed a four-step process that involved identification, elimination of duplicates, relevance screening and full-text eligibility assessment. To begin with, all the acquired papers were tabulated out of the selected databases. The duplicate records were then eliminated by metadata matching and manual verification procedures. Title and abstract screening were used to filter irrelevant publications which are not related to the field of IoT or IIoT intrusion detection datasets. The full-text evaluation was based on the initial screening to obtain methodological relevance, dataset significance, experimental validity, and analytical contribution. The studies were given special attention, and they introduce benchmark datasets, evaluate the generalization of IDS, address the issue of imbalance in the dataset, or discuss explainability, federated learning, temporal modeling, and cross-domain intrusion detection. The taxonomy building, comparative dataset analysis, the realism evaluation, and the bias-oriented evaluation were based on the last set of available studies.

**Figure 4:**
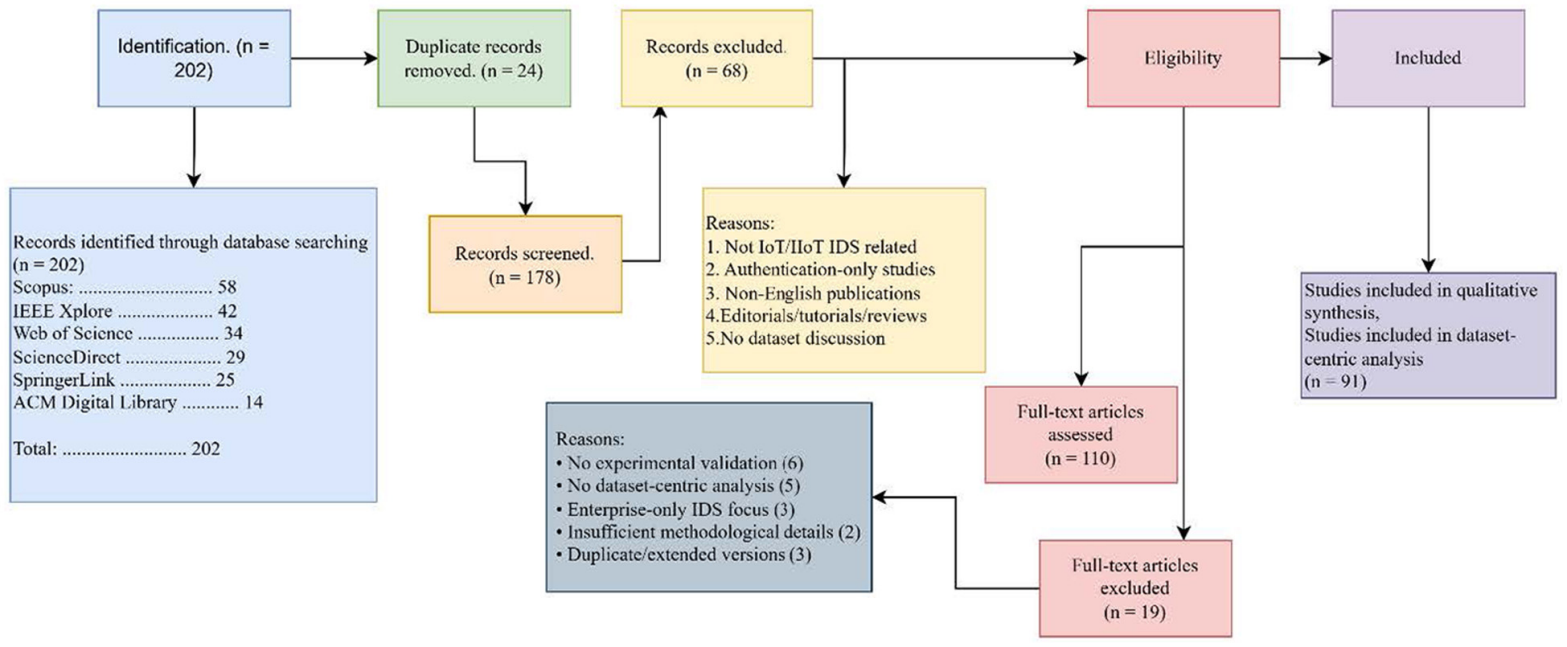
PRISMA-based study selection workflow illustrating literature identification, screening, eligibility assessment, exclusion reasons, and final inclusion of 88 studies used in the dataset-centric analysis.

The screening process followed for the conduct of this review is shown in Figure 4. There were 202 records found in the first search in six major scientific databases. Twenty-four duplicate records were eliminated and 178 studies were screened for title and abstract. After relevance assessment, 110 full-text articles were assessed, with 91 studies meeting all methodological and dataset-centric eligibility criteria. This process provided a transparent, reproducible and methodological review.

The study-selection process used in this review, based on the PRISMA, is summarized in Table 5. Two hundred and two records were first identified from the major scientific databases with 24 duplicate records excluded before screening. Ninety-one studies met the inclusion criteria after eligibility assessment and were included in the dataset-centered comparative analysis.

**Table 5:** PRISMA-based study selection statistics.

Selection stage	Count
Records identified	202
Duplicates removed	24
Records screened	178
Records excluded	68
Full-text assessed	110
Full-text excluded	19
Final studies included	91

#### 4.5.1 Reproducibility statement

The literature search is carried out from January to March 2026. The same Boolean expressions were used to conduct all searches in Scopus, Web of Science, IEEE Xplore, ScienceDirect, SpringerLink, and ACM Digital Library. The duplicate removal was done by the metadata matching and manual verification. The data were extracted based on a template that consisted of dataset name, publication year, traffic representation, protocol coverage, attack diversity, temporal continuity, and evaluation metrics.

### 4.6 Structured data extraction framework

An analytical framework of data extraction was established to guarantee analytical consistency and replication across heterogeneous studies of IDS and benchmark datasets.

In each of the chosen studies, the following characteristics were systematically outlined and noted:

The organized process of extraction made it possible to compare cross-databank and reduced inconsistencies due to heterogeneous evaluation methodologies in Table 6.

**Table 6:** Data extraction structures to be used in comparative analysis of IoT and IIoT intrusion detection data.

Attribute	Description
Dataset name	Benchmark dataset used in evaluation
Domain context	IoT, IIoT, CPS, SDN-IoT, or traditional IT
Traffic representation	Packet-level, flow-level, or hybrid
Protocol semantics	MQTT, CoAP, Modbus, DNP3, OPC-UA, HTTP, etc.
Attack categories	DDoS, botnet, malware, reconnaissance, APT, spoofing
Learning paradigm	ML, DL, FL, XAI, hybrid IDS
Evaluation metrics	Accuracy, F1-score, FAR, Recall, Precision, AUC
Temporal characteristics	Sequential continuity and time dependency
Realism indicators	Device diversity, operational realism, process awareness
Bias indicators	Class imbalance, redundancy, leakage, synthetic traffic
Deployment suitability	Edge computing, federated learning, explainable IDS readiness

### 4.7 Dataset-centric taxonomy construction

To overcome the inconsistencies in the IDS benchmarking practices, the study proposes a single data centric taxonomy that can classify intrusion detection datasets in terms of domain context, traffic representation, protocol specificity, attack realism and learning appropriateness. The taxonomy framework was to be created to address the common practice to treat datasets describing the operational and semantic differences between IoT and IIoT and traditional network intrusion data interchangeably. Databases were classified in the following dimensions of analysis.

This taxonomy allows a systematic comparison between datasets and allows detection of dataset-model mismatches that often yield spurious IDS evaluation results in Table 7.

**Table 7:** Dimensions of taxonomy and analytical purposes of dataset-centric classification of IoT and IIoT intrusion detection datasets.

Taxonomy dimension	Analytical purpose
Application domain	Distinguishes IoT, IIoT, CPS, and enterprise IT contexts
Traffic representation	Determines packet-level or flow-level suitability
Protocol specificity	Evaluates semantic realism of industrial protocols
Temporal structure	Assesses sequential continuity for DL architectures
Threat modeling	Evaluates diversity and realism of attack scenarios
Learning suitability	Determines compatibility with ML/DL/FL/XAI paradigms
Deployment relevance	Assesses edge, cloud, and federated deployment feasibility

### 4.8 Dataset quality evaluation and bias assessment

One of the key contributions of this review is that it introduces a dataset-centric analytical framework as a means of assessing the limitations of realism and methodological bias that are immanent to contemporary IDS datasets. This study measures datasets based on various dimensions of realism and reliability, such as:

- Traffic realism
- Threat diversity
- Protocol coverage
- Temporal continuity
- Label consistency
- Device heterogeneity
- Scalability
- Explainability support
- Federated learning readiness

Systematically, the review examines various types of evaluation bias which may artificially overstate IDS performance claims, such as:

- Class imbalance bias
- Dataset redundancy
- Temporal leakage
- Feature leakage
- Synthetic traffic dominance
- Limited attack variability
- Protocol underrepresentation
- Dataset-model incompatibility

A comparative analysis of cross-datasets was then conducted to address the question of how these biases affect the reported effectiveness, generalization ability and deployment readiness of ML/DL-based intrusion detection systems.

### 4.9 Comparative analytical synthesis

After building taxonomy and characterizing datasets, comparative analytical synthesis was conducted on datasets characterizing the IoT, IIoT, CPS, and traditional network intrusion detection datasets.

The comparative evaluation investigated:

- Disagreements in the behavior of traffic in IoT and IIoT.
- Influence of flow-based over packet-based representations.
- Correlation of complexity of dataset and accuracy of IDS.
- Effects of temporal continuity on sequence-based DL models.
- Impacts of imbalance reduction measures on minority attacks.
- Limitations on generalization to heterogeneous datasets.
- Suitability of datasets to federated and explainable intrusion detection.

Instead of concentrating on algorithmic benchmarking, the synthesis considers the basic role of dataset properties in determining the results of IDS assessments and deployment viability.

### 4.10 Methodological limitations

Even though this review is carried out based on rigorous and systematic methodology, it is necessary to note several limitations. To begin with, the analysis is based on publicly available datasets and published experimental results, which can contain undocumented preprocessing operations, hidden leakage mechanisms, or inconsistent validation protocols. Second, the dynamic, evolving nature of the IoT and IIoT threat landscapes can bring about future categories of attacks, which are not represented in existing benchmark datasets. Third, some more recently suggested datasets have not been adequately tested in large-scale real world industrial conditions. However, the proposed methodology will offer a transparent, reproducible, and analytically based framework to assess the realism, reliability, and deployment relevance of modern IoT and IIoT intrusion detection datasets.

## 5 Dataset classification framework

Current sample survey of intrusion detection data sets is mainly based on descriptive comparisons and reported detection rates to gauge usefulness of dataset. Nevertheless, these methods do not always give objective information about realism, reliability, scalability, and deployment suitability of datasets to implement research on the IoT and IIoT intrusion detection. Consequently, numerous benchmark datasets are still in use despite severe limitations, namely, class imbalance, synthetic traffic dominance, temporal inconsistency, and limited protocol diversity. To overcome these drawbacks, this paper proposes a Quantitative Dataset Scoring Framework, which is a quantitative framework that systematically assesses IoT and IIoT intrusion detection datasets across various analytical dimensions. In contrast to the traditional qualitative reviews, the proposed framework changes the evaluation of datasets into the measurable and reproducible process by assigning weighted scores to critical characteristics of datasets. The suggested framework facilitates:

- Objective comparison between datasets.
- Identification of the limitations of realism.
- Analysis of evaluation biases.
- Evaluation of the real world applications.

Identification of suitable datasets to be used in benchmarking IDS. This framework can also be used to explain why there are always high IDS accuracies with certain datasets even when those datasets have limited real-world representativeness.

### 5.1 Motivation for a dataset-centric classification framework

The rapid proliferation of IoT and IIoT intrusion detection datasets has resulted in a fragmented research landscape in which fundamentally different datasets are often used interchangeably. In this regard, domain context, traffic representation, protocol semantics, and threat realism are merely a few critical differences leading to potentially misleading evaluation outcomes, coupled with inflated performance claims. To this end, we suggest a combined dataset classification framework to categorize IoT and IIoT intrusion detection datasets depending on the domain of application, the representation of traffic, the specificity of protocols, and the focus of attacks. This framework is not a simple organization instrument but a methodical prism with the help of which the suitability of the data set, evaluation bias and deployment relevance can be investigated.

### 5.2 High-level taxonomy of IoT and IIoT IDS datasets

**Figure 5:**
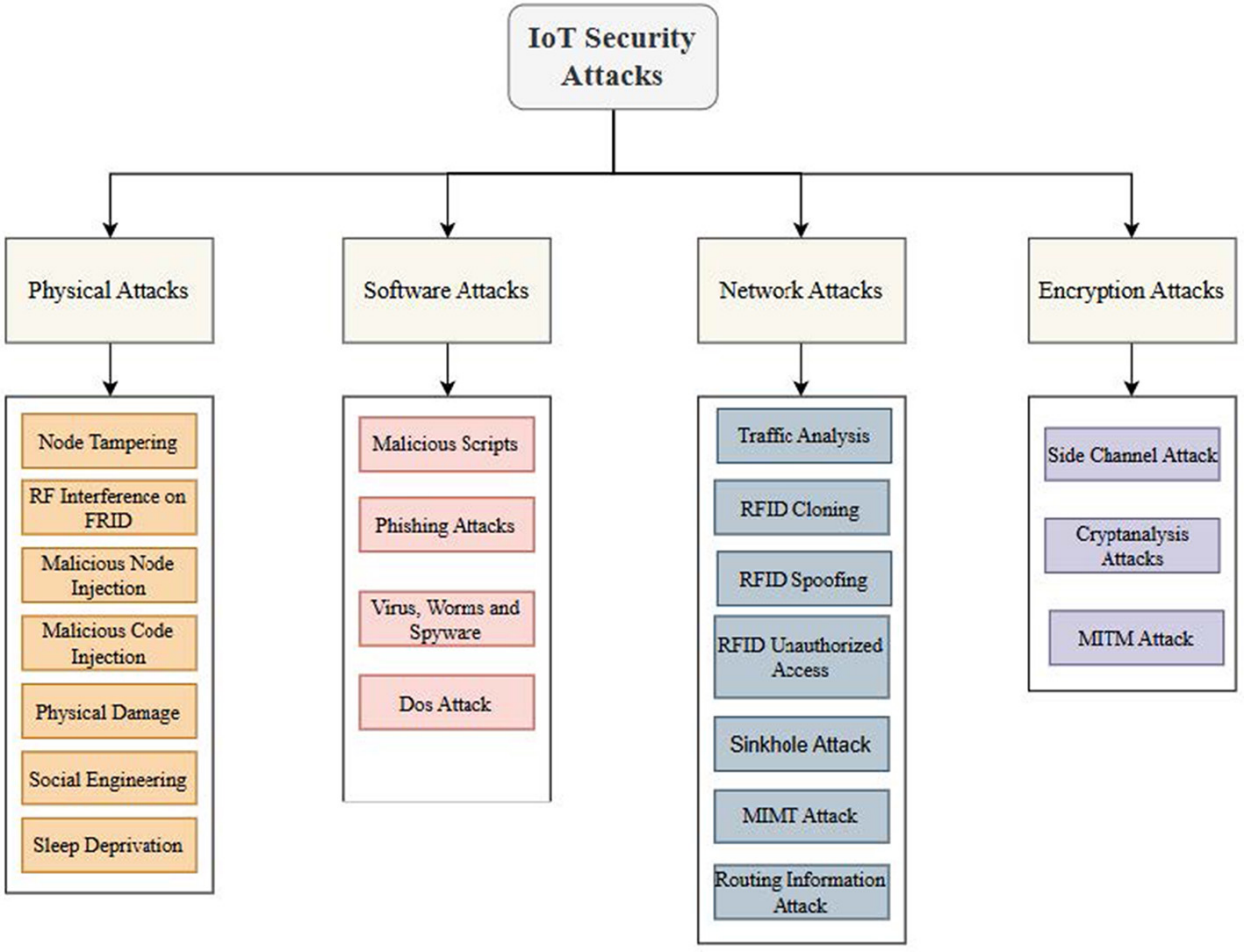
IoT security attacks classification: physical, software, network, and encryption-level attack identification.

Figure 5 illustrates the taxonomy of all the types of IoT security attacks to four broad categories, which are physical, software, network, and encryption attacks. The primary goal of physical and software attacks is to affect the IoT devices and applications through node tampering, code injection, phishing, and denial-of-service attacks that may damage the devices or interrupt the services. Network and encryption attacks are aimed at communication channels and cryptographic programs, such as traffic monitoring, RFID attacks, man in the middle attacks, and cryptanalysis, thereby causing a risk to the confidentiality, integrity, and availability of data in IoT scenarios.

This taxonomy underlines four major dataset categories.

- IIoT and CPS-oriented datasets.
- IoT botnet-focused datasets.
- Flow-based IoT intrusion datasets.
- Protocol-specific and traditional NIDS datasets.

Each represents different design goals and assumptions about its evaluation, as discussed in detail below.

### 5.3 Master dataset categorization

The classification of widely used intrusion detection datasets with respect to the proposed framework is presented in Table 8. This table is used as a reference throughout the rest of the paper to facilitate consistent cross-dataset comparisons and critical analyses.

**Table 8:** Master categorization of IoT and IIoT intrusion detection datasets.

Category	Datasets	Domain focus	Traffic representation	Key characteristics
IIoT/CPS-oriented	Edge-IIoTSet, ToN_IoT, X-IIoTID, SWAT, WADI, WUSTL-IIoT-2021, and CICAPTIIoT	Industrial automation, CPS	Packet/flow + process context	Protocol-aware, temporal, and safety-critical
IoT botnet-focused	BoT-IoT, N-BaIoT, and MeDBoT	Consumer IoT	Flow-based	High attack volume, severe imbalance
IoT flow-based	IoTID20, CIC-IoT23, and IoT-23	General IoT	Flow-based	Scalable, encrypted-traffic compatible
Protocol-specific	MQTT-IoT/MQTT Set	IoT messaging	Packet/flow	Deep protocol semantics
Traditional NIDS	CIC-IDS-2017, CSE-CIC-IDS-2018, and NSL-KDD	Enterprise IT	Flow/feature-based	Legacy traffic patterns
DDoS-focused	CIC-DDoS2019, IoT DoS/DDoS	IoT and IT	Flow-based	High-rate volumetric attacks

### 5.4 Why IIoT datasets like SWAT/WADI are fundamentally different from BoT-IoT

A common pitfall in IDS research is the interchangeable use of IIoT and IoT datasets. Datasets such as SWAT and WADI are representative of an industrial control environment, wherein network traffic is tightly coupled with physical process behavior. Most of the attacks in these datasets manifest more often as subtle deviations in the control logic or sensor readings, not necessarily as abrupt traffic anomalies. In contrast, the BoT-IoT and similar datasets focus on high-volume botnet activities that target consumer IoT devices. These types of attacks are usually easier to detect because they are volumetric in nature and repetitive in pattern. The result is that most models, when trained using IoT botnet datasets, report extremely high accuracy but often fail in IIoT environments, where attacks are stealthier and depend on context. This distinction underlines that no performance results obtained on IoT botnet datasets can be directly extrapolated to the security scenarios of the IIoT or CPS.

### 5.5 Flow-based vs. packet-based datasets: implications for IDS evaluation

Traffic representation plays a decisive role in dataset usability and model selection. Packet-based datasets retain finer-grained protocol interactions and payload data; therefore, they serve deep protocol analysis and signature-based detection. They are, however, computationally costly and not as compatible with encrypted traffic. Flow-based datasets containing time-window aggregated traffic statistics are scalable and resistant to encryption. Thus, most IDS studies in recent times are based on flow-based data. Nonetheless, flow aggregation can conceal low-rate or sleek attacks, particularly in IIoT systems, in which malicious traffic can look very similar to legitimate operational traffic. Therefore, the choice between packet-based and flow-based datasets reflects a trade-off between detection granularity and deployment feasibility, which must be explicitly acknowledged in the evaluations of IDS.

### 5.6 Why protocol-specific datasets are important

Protocol-specific datasets, such as MQTT-focused corpora, constitute fundamental building blocks necessary for filling the gap in IDS research to capture semantics at the application layer. IoT protocols often have specific communication patterns, quality-of-service mechanisms, and control messages that directly influence system behavior. Neglecting protocol semantics leads to IDS models that detect statistical anomalies without attempting to capture the malicious intent. Protocol-specific datasets are thus imperative to facilitate further advances in context-aware intrusion detection, and they cannot be treated as interchangeable but are complementary to general-purpose IDS datasets.

### 5.7 Traditional NIDS datasets in IoT and IIoT research

Traditionally, intrusion detection datasets include CIC-IDS-2017 and NSL-KDD, which are still baseline benchmark datasets for many researchers because they have easily accessible structured feature sets. These datasets were designed for IT networks within an enterprise and do not capture the key features of IoT/IIoT, including lightweight protocols, device heterogeneity, and real-time constraints.

Such datasets can provide value for algorithmic benchmarking; however, their limitations should be clearly acknowledged when interpreting the claims of IDS performance in IoT and IIoT contexts.

### 5.8 Quantitative dataset scoring framework

#### 5.8.1 Motivation

While existing surveys often compare intrusion datasets based on descriptive characteristics, these comparisons often do not have objective criteria for assessing intrusion dataset quality and deployment suitability. Thus, datasets that contain significant shortcomings, such as class imbalance, lack of protocol variety, synthetic traffic dominance, and poor temporal continuity, can still be utilized in benchmarking studies of IDS. To overcome this, a score system for quantitative evaluation of IoT, IIoT, and traditional IDS datasets is proposed. The framework transforms qualitative features of a dataset to numeric scores, allowing comparison of the benchmark datasets and identifying the most appropriate one for realistic evaluation of intrusion detection systems.

#### 5.8.2 Dataset evaluation dimensions

The scoring framework evaluates each dataset using eight critical dimensions identified through the systematic review.

Table 9 provides a list of the dimensions for the evaluation as outlined in the proposed scoring framework. The weight given to traffic realism is the highest, as realistic behavior of the network will directly influence the validity of IDS deployment. Threat diversity is given high importance as it affects attack representativeness; protocol coverage is also important, and temporal continuity is also important to affect the effectiveness of the modern ML/DL-based IDS models. Explainability support and federated learning readiness are added to capture some of the requirements of next-generation intrusion detection systems.

**Table 9:** Quantitative dataset evaluation dimensions and weights.

Dimension	Description	Weight (%)
Traffic realism	Degree to which traffic reflects real-world operational behavior	20
Threat diversity	Variety of attack categories represented	15
Protocol coverage	Coverage of IoT/IIoT communication protocols	15
Temporal continuity	Availability of sequential and time-dependent traffic patterns	15
Device heterogeneity	Diversity of devices and network entities	10
Explainability support	Availability of interpretable features and annotations	10
Federated learning readiness	Suitability for distributed learning environments	10
Deployment suitability	Applicability to real-world operational environments	5
**Total**		**100**

#### 5.8.3 Quantitative scoring methodology

The five dimensions were assessed in each of the data sets on a scale of 1 (Very Poor) to 5 (Excellent). A weighted sum of the individual dimension scores was then calculated to get an overall score for the dataset, which quantitatively measures the quality of the dataset, realism, and deployment suitability.

Dataset scoring equation:


$$\begin{array}{l} DS=\overset{n}{\underset{i=1}{\sum }}w_{i}S_{i} \end{array}$$ (1)

Where:

- DS, overall dataset score;
- Si, score assigned to criterion *i*;
- wi, weight assigned to criterion *i*;
- *n* = number of evaluation criteria.

This score is then scaled from 0 to 100. The scoring Equation (1) gives a clear and repeatable way of assessing datasets. The higher the scores the more realistic the datasets, the more fully the datasets cover protocols, the better the temporal properties, and the more suitable the deployment. The evaluation results for comparative datasets are displayed here.

#### 5.8.4 Comparative dataset evaluation results

The comparative evaluation is used to highlight the significant differences in the quality of the datasets and in their relevance for deployment in Table 10. The scores of Edge-IIoTSet and ToN-IoT are high due to realistic traffic generation, a variety of attack scenarios, protocol richness, and high deployment relevance. The performance of X-IIoT and WUSTL-IIoT is also good, because of the industrial communication characteristics and the temporal consistency. Legacy datasets (NSL-KDD and CIC-DDoS2019) get lower scores due to the absence of modern IoT protocols, realistic attack behavior, and modern attack diversity. The results indicate that the realism and suitability of the dataset for deployment should be considered in addition to the accuracy of IDSs reported in the literature when selecting benchmark datasets.

**Table 10:** Quantitative scores of representative IoT, IIoT, and traditional IDS datasets.

Dataset	Score (/100)
Edge-IIoTSet	89
ToN-IoT	86
X-IIoTID	82
WUSTL-IIoT	80
CICAPTIIoT	78
CIC-IoT23	76
MQTT-IoT	72
IoTID20	70
BoT-IoT	64
N-BaIoT	61
CIC-IDS2017	66
UNSW-NB15	63
CIC-DDoS2019	60
IoT-DoS/DDoS	58
NSL-KDD	52

A moderate performance is obtained for IoT datasets, which have greater attack diversity, though they also might have class imbalance and limited industrial protocol coverage. Traditional IDS datasets are still useful for baseline benchmarking, but they are not well-suited for the next generation of intrusion detection research due to their outdated nature and poor representation of modern IoT and IIoT environments.

## 6 Detailed dataset-wise review

### 6.1 Industrial Internet of Things (IIoT) datasets

#### 6.1.1 Edge-IIoTSet

Edge-IIoTSet (Ferrag et al., 2022) is a complete Industrial IoT dataset designed to model realistic smart manufacturing and edge computing environments. This was generated through a controlled IIoT testbed that integrated sensors, edge devices, and cloud services, thereby capturing both benign and malicious traffic. It includes flow-based and packet-level traffic across various industrial and IoT protocols, such as MQTT, Modbus, and TCP/IP. A wide range of attacks, such as DoS, DDoS, reconnaissance, injection, and malware-based intrusions, were included. Because it is large-scale with protocol diversity, Edge-IIoTSet is widely applied for the evaluation of machine learning and deep learning-based IDS models in industrial edge environments.

#### 6.1.2 . ToN_IoT

The ToN_IoT (Moustafa et al., 2020) dataset was designed to represent the concept of a hybrid IoT–IIoT–cloud ecosystem by combining network traffic with system logs and telemetry data. It contains realistic attack scenarios for IoT devices, cloud services, and industrial components. ToN_IoT incorporates multiple types of attacks, such as DDoS, backdoor, password, injection, and man-in-the-middle attacks. In addition, ToN_IoT supports multimodal intrusion detection research by providing various heterogeneous data sources. Its complexity makes the dataset suitable for evaluating advanced deep, ensemble, and federated learning-based IDS approaches.

#### 6.1.3 X-IIoTID

X-IIoTID (Al-Hawawreh et al., 2021) is an Industrial IoT intrusion detection dataset that mainly targets industrial control system communication patterns. The traffic to be analyzed was at the packet level and generated from realistic IIoT and ICS environments using protocols such as Modbus and Ethernet/IP. The dataset includes attacks such as command injection, replay attacks, and DoS against the control processes. X-IIoTID focuses on protocol-aware intrusion detection and is usually used in fine-grained analyses related to vulnerabilities in industrial communications. Its structured attack labeling makes it suitable for supervised ML- and DL-based IDS evaluations.

#### 6.1.4 WUSTL-IIoT-2021

WUSTL-IIoT-2021 (Zolanvari et al., 2023) is a newly developed industrial IoT dataset that emulates cyber-physical system traffic in industrial settings. The focus of the dataset is on flow-based network traffic, which includes both usual operations and several attack scenarios, such as spoofing, reconnaissance, DoS, and DDoS. The dataset was generated in realistic industrial automation settings and reflected typical IIoT communication patterns. Its moderate size and well-defined features make it suitable for benchmarking lightweight IDS models in this study. WUSTL-IIoT-2021 is typically employed in comparison with classical ML and hybrid IDS techniques.

#### 6.1.5 . CICAPT-IIoT

CICAPTIIoT (Ghiasvand et al., 2024) is a modern IIoT dataset developed to model advanced and persistent threats in industrial environments. It captures complex multistage attack behaviors, such as lateral movement, privilege escalation, data exfiltration, and coordinated DDoS attacks. This is a flow-based dataset that contains rich feature representations extracted using the CICFlowMeter tool. CICAPTIIoT is particularly significant when assessing advanced IDS solutions against stealthy and long-duration attacks. Its realistic threat modeling is well suited to next-generation industrial cybersecurity research.

### 6.2 Internet of Things IoT datasets

#### 6.2.1 BoT-IoT

The BoT-IoT (Koroniotis et al., 2019) is a large-scale IoT botnet dataset built to simulate realistic botnet-driven attacks in IoT networks. Both packet-level and flow-based traffic are included in the dataset, which considers different types of attacks, such as DDoS, DoS, reconnaissance, and data exfiltration. The dataset was generated in a controlled environment in which numerous compromised IoT devices communicated with command-and-control servers. Owing to its colossal size and high volume of attack traffic, BoT-IoT is widely adopted in deep learning-based IDS research. It is effective for studying the behavior of botnets and detecting large-scale attacks.

#### 6.2.2 N-BaIoT

N-BaIoT (Meidan et al., 2018) is an IoT botnet detection model based on the traffic generated by infected consumer IoT devices. It primarily comprises packet-level traffic and focuses on variants of the Mirai and BASHLITE botnets. The dataset provides statistical features extracted from network traffic streams, allowing device behavior-based intrusion detection. N-BaIoT is especially appropriate in anomaly detection and deep autoencoder-based IDS technology. Moreover, its machine-based design renders it very applicable in the research of light and edge-based Internet of Things (IoT) security solutions.

#### 6.2.3 IoT-23

IoT-23 (Sharma and Babbar, 2024) is a packet-capture based data, which consists of the actual and simulated malware traffic of the IoT in the honeypots and controlled settings. It addresses the benign IoT communications as well as malicious ones, including botnet infections, command and control communications, and data exfiltration. In contrast to feature-engineered datasets, the IoT-23 introduces raw PCAP files, and this allows researchers to achieve latitude to extract custom features. This flexibility renders it sufficient to protocol level analysis and 0-day attacks analysis. IoT-23 has been used in the studies of malware traffic classification.

#### 6.2.4 IoTID20

IoTID20 (Ullah and Mahmoud, 2020) is a flow-based IoT intrusion data obtained with the help of a realistic IoT network environment. It contains both normal traffic and various forms of attacks, which include DoS, DDoS, Mirai botnet attacks, scanning attacks and man-in-the-middle attacks. Both classical machine learning and deep learning models are appropriate to the rich set of statistical flow features of the data. The benchmark of IoT-specific environment IDS is mostly done by IoTID20. It has a balance structure that can be applied in binary as well as multi-class classification.

#### 6.2.5 MQTT-IoT/MQTT set

The MQTT-IoT (Vaccari et al., 2020) dataset focuses on security threats targeting the MQTT protocol, which is a widely adopted protocol for communications in IoT. This dataset includes packet-level traffic that captures both normal MQTT operations and malicious activities, such as flooding attacks, unauthorized access, and the injection of malformed messages. This allows for protocol-specific intrusion detection studies. Specifically, MQTT-IoT is helpful for evaluating lightweight IDS solutions to be deployed on resource-constrained IoT devices. It is important to study vulnerabilities in publish-subscribe-based IoT systems.

#### 6.2.6 CIC-IoT23

This is a modern, large-scale IoT dataset proposed by the Canadian Institute for Cybersecurity to represent recent IoT threat landscapes. It consists of flow-based traffic generated from a wide variety of IoT devices running under both benign and malicious conditions. The set includes attacks against IoT, such as DDoS, malware propagation, botnet activities, and web-based attacks. CIC-IoT23 (Maniriho et al., 2020) features a rich set of extracted features using standardized tools, making it suitable for reproducible IDS studies. It is regarded as a state-of-the-art benchmark for evaluating next-generation IoT intrusion detection systems.

### 6.3 Intrusion Detection System (IDS)-related datasets

#### 6.3.1 CIC-IDS-2017

CIC-IDS-2017 (Panigrahi and Borah, 2018) is one of the most commonly used benchmark datasets in network intrusion detection research. It features a realistic enterprise network environment with benign traffic and various attack scenarios executed over successive days. The newer types of attacks in this dataset include DDoS, DoS, port scan, botnet, web-based, and brute-force attacks. This is flow-based traffic with several rich statistical features extracted using the CICFlowMeter tool. Owing to its realism and comprehensive coverage of attacks, CIC-IDS-2017 has been extensively used to evaluate machine learning and deep learning-based IDS models. However, this dataset faces issues related to class imbalance, making the detection of minority attacks difficult.

#### 6.3.2 UNSW-NB15

UNSW-NB15 (Moustafa and Slay, 2015) was created to address the weaknesses of old IDS datasets by incorporating the modern attack behavior and realistic network traffic. The dataset has nine significant categories of attackers, such as fuzzers, exploits, reconnaissance, shellcode, and denial of service attack. The characteristics of this dataset are flow-based and are obtained out of raw packet captures through the newest traffic analysis tools. UNSWNB15 is more balanced in terms of normal and malicious traffic when compared with previous benchmarks. UNSW-NB15 is commonly used with traditional machine learning algorithms and hybrid deep learning models in terms of performance assessment. Some of the attack patterns in UNSWNB15 are fairly easy to understand despite several benefits in comparison to the contemporary IoT/IIoT attacks.

#### 6.3.3 NSL-KDD

NSL-KDD is an enhanced version of the KDD Cup (Tavallaee et al., 2009) 1999 dataset, which was built to eliminate redundant records and reduce the risk of a biased evaluation. It includes four major categories of attacks: denial of service, probing, remote-to-local, and user-to-root attacks. It is feature-based instead of flow- or packet-based, which makes it lightweight and easy to handle in computations. Owing to its simplicity and broad acceptance, it is still used to perform various benchmarking and comparative analyses. However, it cannot represent advanced cyber threats or modern network traffic. Consequently, it is not well-applicable to contemporary IoT and IIoT environments.

#### 6.3.4 CIC-DDoS2019

CIC-DDoS2019 (Sharafaldin et al., 2019) is a domain-specific dataset that focuses only on DDoS attacks. It features an impressive variety of high-impact DDoS attack types, such as SYN floods, UDP floods, LDAP amplification, MSSQL attacks, and NetBIOS-based attacks. Data were generated in a realistic network environment and consisted of flow-based traffic with an extensive number of statistical features. In particular, the CIC-DDoS2019 dataset is suitable for fine-grained research on DDoS detection and classification. Its large scale allows for good evaluations of deep learning models. However, this dataset lacks benign application diversity beyond DDoS scenarios.

#### 6.3.5 IoT DoS/DDoS dataset

The IoT DoS/DDoS (Hussain et al., 2020) dataset was created to capture DoS attacks against IoT networks and resource-constrained devices. It contains packet-level traffic that characterizes both normal IoT communications and several DoS/DDoS flooding-based attack types. The dataset reflects the most common attack strategies against IoT devices, including TCP, UDP, and HTTP flooding. This dataset is beneficial for the performance evaluation of lightweight IDS solutions deployed at the IoT edge. Because this dataset is focused on DoS-related threats, it is most often used in binary and multiclass classification tasks. This dataset is mainly limited to only a few attack types, which limits its use in general-purpose intrusion detection.

## 7 Comparative analysis of IoT and IIoT IDS datasets

### 7.1 Cross-dataset comparison

Table 11 illustrates the realism, attack diversity, class imbalance severity, and protocol depth of representative IoT, IIoT, and traditional IDS datasets. These dimensions affect the validity of IDS performance evaluation and their readiness for deployment.

**Table 11:** Comparative assessment of IoT and IIoT IDS datasets based on realism, temporal continuity, Encrypted traffic support, and deployment suitability.

Dataset	Domain	Realism	Attack diversity	Imbalance severity	Temporal continuity	Encrypted traffic support	Deployment suitability
Edge-IIoTSet (Ferrag et al., 2022)	IIoT	High	High	High	Medium	Partial	Excellent
ToN_IoT (Moustafa et al., 2020)	IoT/IIoT	Medium–High	High	Medium–high	Medium	Partial	Excellent
X-IIoTID (Al-Hawawreh et al., 2021)	IIoT	Medium	Medium	Medium	High	Partial	Excellent
SWAT (Rapousis et al., 2015)	CPS/IIoT	High	Low–medium	Medium	High	No	Excellent
WADI (Ahmed et al., 2017)	CPS/IIoT	High	Medium	Medium	High	No	Excellent
BoT-IoT (Koroniotis et al., 2019)	IoT	Low	Medium	**Extreme**	Low	No	Moderate
N-BaIoT (Meidan et al., 2018)	IoT	Low–medium	Low	High	Low	No	Moderate
IoTID20 (Ullah and Mahmoud, 2020)	IoT	Medium	Medium	Medium	Low	No	Moderate
MQTT-IoT (Vaccari et al., 2020)	IoT	Medium	Low	Medium	Medium	Partial	Good
CIC-IoT23 (Maniriho et al., 2020)	IoT	Medium	High	High	Medium	Partial	Good
CIC-IDS-2017 (Panigrahi and Borah, 2018)	Traditional IT	Medium	High	High	Low	No	Moderate
NSL-KDD (Tavallaee et al., 2009)	Traditional IT	Low	Low	Medium	Low	No	Poor
CIC-DDoS2019 (Sharafaldin et al., 2019)	IT/IoT	Medium	Low	High	Low	No	Moderate
IoT DoS/DDoS (Hussain et al., 2020)	IoT	Low–medium	Low	High	Low	No	Moderate

The bold value (“Extreme”) indicates means the high level of class imbalance compared to other datasets.

Key observations:

- Most IIoT and CPS datasets focus on realism and protocol semantics with limited attack diversity.
- IoT botnet datasets focus on attack volume rather than on behavioral realism.
- Traditional IDS datasets remain popular but lack IoT-specific characteristics.
- Protocol-specific datasets provide deep semantic insights but have limited generalizability.

This comparison underscores the reasons why dataset selection fundamentally constrains IDS evaluation outcomes.

Table 12 displays a detailed comparison of popular intrusion detection datasets that were released from 2009 to 2024, both for the IoT and IIoT domains, along with those of conventional IDS datasets. The datasets vary widely in size (from thousands to millions of rows), feature dimensionality, type of traffic, domain, and types of attacks. Several recent datasets are created for realistic attack scenarios in the industrial IoT (IIoT), including Edge-IIoTSet, X-IIoTID, and CICAPTIIoT, while others are designed to address large scale threats in IoT botnets, for example, BoT-IoT, IoT-23, and N-BaIoT. CIC-IDS2017, UNSW-NB15, and NSL-KDD are still very popular benchmarks used for evaluating and comparing intrusion detection models in different cyber security scenarios.

**Table 12:** Comparative analysis of IIoT, IoT, and IDS datasets.

Dataset	Year	Domain	Traffic type	No. of features	Attack types	Dataset size (approx.)
Edge-IIoTSet (Ferrag et al., 2022)	2022	IIoT	Flow + packet	61	DoS, DDoS, reconnaissance, injection, malware, and brute-force	~14 million flows
ToN_IoT (Moustafa et al., 2020)	2020	IoT/IIoT/Cloud	Flow + telemetry	Not specified	DDoS, backdoor, injection, and MITM, password attacks	~9 million records
X-IIoTID (Al-Hawawreh et al., 2021)	2021	IIoT/ICS	Packet-based	68	DoS, command injection, and replay	~1.5 million packets
WUSTL-IIoT-2021 (Zolanvari et al., 2023)	2021	IIoT	Flow-based	41–48	DoS, DDoS, spoofing, and reconnaissance	~800k flows
CICAPTIIoT (Ghiasvand et al., 2024)	2023	IIoT	Flow-based	32	APT, DDoS, lateral movement, injection	~3.5 million flows
BoT-IoT (Koroniotis et al., 2019)	2018	IoT Botnet	Packet + flow	46	DDoS, DoS, reconnaissance, and data exfiltration	~72 million records
N-BaIoT (Meidan et al., 2018)	2018	IoT Botnet	Packet-based	115	Mirai, BASHLITE botnet attacks	~7 million packets
IoT-23 (Sharma and Babbar, 2024)	2020	IoT	Packet-based	23	Botnet, C&C, and malware	~20 million packets
IoTID20 (Ullah and Mahmoud, 2020)	2020	IoT	Flow-based	83	DoS, DDoS, mirai, scan, and MITM	~625k flows
MQTT-IoT (MQTT set) (Vaccari et al., 2020)	2021	IoT (protocol-specific)	Packet-based	~67	MQTT flood, unauthorized access, and DoS	~1 million packets
CIC-IoT23 (Maniriho et al., 2020)	2023	IoT	Flow-based	78	DDoS, malware, botnet, web attacks	~5 million flows
CIC-IDS-2017 (Panigrahi and Borah, 2018)	2017	Traditional NIDS	Flow-based	78	DDoS, DoS, PortScan, bot, and web attacks	~3 million flows
UNSW-NB15 (Moustafa and Slay, 2015)	2015	Traditional NIDS	Flow-based	49	Fuzzers, exploits, DoS, and reconnaissance	~2.5 million records
NSL-KDD (Tavallaee et al., 2009)	2009	Traditional NIDS	Feature-based	41	Probe, DoS, R2L, U2R	~148k records
CIC-DDoS2019 (Sharafaldin et al., 2019)	2019	DDoS-focused	Flow-based	88	SYN flood, UDP flood, LDAP, and MSSQL DDoS	~12 million flows

Table 13 shows the results of many studies that obtain above 99% accuracy but in fact remain on datasets where the classes are not balanced, the attacks are not dominant, or where balancing methods are used before training the model. Thus, high reported accuracy must be taken with some healthy skepticism for being equivalent in heterogeneous real-world deployment environments in IoT and IIoT.

**Table 13:** Evidence supporting the influence of dataset characteristics on reported IDS performance.

Dataset	Evidence of imbalance/dataset characteristics	Reported Accuracy
BoT-IoT (Luqman et al., 2025)	Authors explicitly balanced 11 classes before training to reduce overfitting and improve generalization	99.60% (RF), 99.97% (LSTM)
N-BaIoT (Sakthipriya et al., 2023)	IoT botnet dataset with high-dimensional traffic requiring dimensionality reduction to improve detection performance	95.02%
CIC-IDS2017 (Osa et al., 2024)	Researchers applied SMOTE and random sampling to address class imbalance before training	99.68%
Edge-IIoTSet (Alatewish, 2025)	Multi-class IDS evaluation achieving very high performance on benchmark dataset	99.97%
IoTID20 (Qaddos et al., 2024)	Authors explicitly state that IoTID20 is highly imbalanced and therefore FW-SMOTE was applied	99.60%
MQTT-IoT IDS2020 (Solanki and Gupta, 2025)	Ensemble IDS achieved very high performance on MQTT benchmark traffic	99.80% (binary), 99.59% (multi-class)
CIC-DDoS2019 (Kapil, 2024)	Random Forest achieved extremely high detection accuracy on DDoS benchmark dataset	99.91%
CICIoT2023 (Almahaqeri et al., 2026)	Authors discuss severe class imbalance and use stratified sampling and feature selection	99.61%

### 7.2 Performance trends and the accuracy illusion

A remarkable trend within the IDS literature is the recurrent reporting of close-to-perfect detection accuracy on some datasets, whereas the performance reported for others is significantly lower. This difference is not primarily algorithmic but rather dataset-driven.

#### 7.2.1 Why BoT-IoT and similar datasets yield ~99% accuracy

Datasets such as BoT-IoT are characterized by:

- Excessive class imbalance.
- Repetitive, high-volume attack patterns.
- Clear statistical separation between benign and malicious traffic.

In these environments, IDS models readily learn to pick up on dataset-specific artifacts rather than actual malicious behavior; even simple classifiers thus achieve extremely high accuracies that create false illusions of security effectiveness.

#### 7.2.2 Why SWAT and WADI report 85%−92% accuracy

CPS-oriented datasets, such as SWAT and WADI, are characterized by:

- Strong coupling between network traffic and physical processes.
- Subtle attack manifestations that resemble normal operational deviations.
- Lower attack-to-normal separability.

Consequently, their detection tasks for IDS models are more realistic; hence, the performance metrics are lower but meaningful. These datasets better reflect the real-world conditions of IIoT security, where the attacks are stealthy and context-dependent.

Accuracy ≠ Security

Unfortunately, high detection accuracy does not imply robust intrusion detection. In most cases, it reflects the following:

- Dataset simplicity.
- Imbalance exploitation.
- Overfitting to static attack patterns.

Therefore, IDS evaluation needs to look beyond aggregate accuracy metrics toward dataset-aware interpretation of results, with the incorporation of realism, temporal behavior, and deployment constraints.

### 7.3 Common evaluation pitfalls in dataset-driven IDS research

Although different types of datasets are available, several evaluation practices undermine the credibility of IDS research.

#### 7.3.1 Data leakage

Feature extraction performed prior to splitting the dataset often results in subtle data leakage, which allows models to learn information that is not available at deployment. This leads to overly optimistic performance estimates.

#### 7.3.2 Random split bias

Random train-test splits do not consider temporal dependencies in network traffic. In an operational environment, IDS models must detect future attacks based on past observations, which current evaluation protocols rarely consider.

#### 7.3.3 Lack of cross-dataset evaluation

Most works assess the IDS models with the help of one dataset, which presupposes generalization. Nevertheless, the models that have been trained on a single dataset tend to be poor when evaluated on other datasets hence revealing significant reliance on the dataset.

#### 7.3.4 Binary vs. multi-class bias

Binary classification systems lower intrusion detections to one malicious class having various attack behaviors. This clouds the under-performance of the poor performance in detecting rare or convoluted attacks.

### 7.4 Comparative analysis key takeaway

Comparing and contrasting all these studies, one will understand that the reported performance of the IDS cannot be discussed without references to the characteristics of the dataset. High-realism and protocol-fidelity datasets have significantly higher detection challenges than high-attack benchmarks. Hence, it is the selection of dataset and evaluation strategy which often has a bigger impact on the reported results than the detection model itself.

### 7.5 Authors vs. accuracy analysis

**Figure 6:**
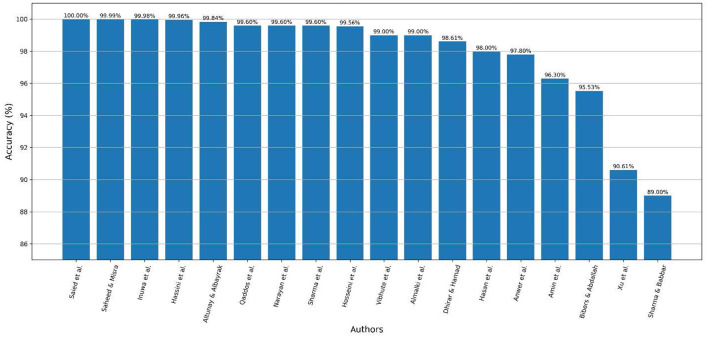
Authors vs. accuracy based on literature review.

Figure 6 is a comparative visualization of authors and intrusion detection accuracy depending on the reviewed literature on cybersecurity in IoT and IIoT. The figure depicts a trend in the performance of different machine learning, deep learning, federated learning and explainable AI-based IDS models on various benchmark datasets. The *x*-axis will show the authors and respective IDS studies, whereas the *y*-axis will indicate the reported intrusion detection accuracy in percentage. The visualization shows that most modern IDS systems are capable of very high detection rates, which are usually over 95, which points to the efficiency of recent intelligent intrusion detection systems. According to Saied et al. the highest accuracy of 100.00 was followed closely by Saheed and Misra with 99.99 and Inuwa et al. with 99.98. Likewise, the studies by Hassini et al. and Altunay and Albayrak reported accuracies of over 99.8, which shows good performance of deep learning-based intrusion detection models using a combination of two or more models. A number of studies such as those by Qaddos et al., Narayan et al., and Sharma et al. have consistently reported about 99.6% accuracy, which demonstrates consistent IDS performance on heterogeneous datasets. Dhirar and Hamad have an accuracy of 98.61, whereas Hasan et al. and Anwer et al. have an accuracy of 98% and 97.8%, respectively. Relatively lower accuracies were reported in works by Xu et al. (90.61%) and Sharma and Babbar (89%), maybe because more complex datasets and realistic attack distributions were used or stricter evaluation protocols were applied. In general, the figure indicates that the modern IDS frameworks always deliver high detection performance in IoT as well as IIoT settings. Nevertheless, the fact that extremely high accuracies are highly concentrated also indicates the possibility of the influence of the dataset imbalance, the production of fake traffic, the redundancy of features and the leakage of time, which can artificially inflate the results of the IDS evaluation.

The performance values listed in Figure 6 come from various studies that use different datasets, binary and multi-class classification, and different preprocessing, feature engineering, and evaluation protocols. It is important to note that the number is only intended to give a comparative overview of reported intrusion detection performance and should not be considered as a direct ranking of the IDS models. For each study, detailed contextual information, such as the dataset used, the type of classification, the metric for evaluation, and reported performance, is given in Table 14. To aid in the proper interpretation of Figure 6 and to accommodate methodological differences between studies, the data set employed, the classification type, the evaluation metric, and reported performance are summarized in Table 14.

**Table 14:** Contextual information supporting (Figure 6).

References	Dataset	Class type	Metric	Performance (%)
Saied et al. (2023)	IoT botnet dataset	Multi	Accuracy	100.00
Saheed and Misra (2025)	Edge-IIoTset	Binary and multi	Accuracy	99.99
Inuwa and Das (2026)	ToN-IoT	Binary	Accuracy	99.98
Hassini et al. (2024)	Edge-IIoTset	Multi	Accuracy	99.96
Altunay and Albayrak (2023)	X-IIoTID	Binary/multi	Accuracy	99.84
Qaddos et al. (2024)	IoTID20	Binary/multi	Accuracy	99.60
Narayan et al. (2024)	NSL-KDD	Multi	Accuracy	99.60
Sharma et al. (2025)	N-BaIoT	Binary/multi	Accuracy,	99.60
Hosseini et al. (2025)	NSL-KDD	Multi	Accuracy	99.56
Vibhute et al. (2024a)	UNSW-NB15	Binary	Accuracy	99.00
Almalki et al. (2025)	BoT-IoT 2020	Binary	Accuracy	99.00
Dhirar and Hamad (2025)	ToN-IoT	Multi	Accuracy	98.61
Hasan et al. (2025)	CIC-DDoS2019	Multi	Accuracy	98.00
Anwer et al. (2025)	X-IIoTID	Multi	Accuracy	97.80
Amin et al. (2025)	CICIDS2017	Binary	Detection accuracy	96.30
Bibers and Abdallah (2025)	N-BaIoT	Binary	Accuracy	95.53
Xu et al. (2021)	NSL-KDD	Binary	Accuracy	90.61
Sharma and Babbar (2024)	BoT-IoT	Binary	Accuracy	89.00

## 8 Open challenges and research gaps in IoT and IIoT IDS datasets

Despite the availability of a few intrusion detection datasets, there is a major constraint in developing viable and deployable IDS solutions. These issues are essentially data-prone in nature and have a direct impact on the accuracy of the reported performance of the IDS.

### 8.1 Lack of 0-day and adaptive attack representation

The available datasets on IoT and IIoT intrusion detection concentrate on the defined and scripted-attack cases. Though these attacks are helpful in supervised learning, they do not capture adaptive adversarial behavior, multi-stage attack campaign and threats that had not been seen before. IDS models are usually learned on such datasets and not the generalized attributes of the intrusions.

Consequence:

Despite the good performance of these solutions, when it comes to generalization to 0-day attacks, their ability to respond to known attack classes is poor and limits their ability to be used in real-world settings, where the threat behavior is constantly evolving.

### 8.2 Encryption blindness in network traffic datasets

The growing use of encryption protocols in communication in IoT/IIoT has made the payload-based inspection ineffective. Nonetheless, a good deal of available datasets is based on unencrypted traffic, and some of them do not consider encrypted patterns of communication, which creates a disparity between dataset and experiment assessments.

Consequence:

IDS models that are trained on plain text traffic do not perform well under the conditions of the real world where encrypted networks are being observed, which normally results in excessively high security guarantees.

### 8.3 Incompatibility with federated and distributed learning paradigms

New paradigms of IDS are increasingly based on federated learning and distributed intelligence to overcome such problems as data privacy, scalability, and other limitations of the regulations. Nevertheless, existing datasets are largely centralized, static, and collected in one administrative domain which offers little to no support of decentralized learning scenarios.

Consequence:

The absence of federated-ready datasets is a severe limitation to additional progress toward privacy preserving and scalable IDS solutions as researchers must depend not on realistic but on synthetic assumptions when evaluating the framework.

### 8.4 Lack of temporal evolution and long-term behavioral context

Most intrusion detection datasets are generated as a snapshot of network traffic and a small number of those have a temporal continuity over extended periods. The attacks are usually injected without a gradual increase in the activities and persistence, and this makes the models unable to acquire temporal dependencies and evolution of behaviors.

Consequence:

The reason is that the LSTM- and transformer-based IDS are trained on non-representative data of the applications, resulting in false inferences on whether they can be applied in the real-life intrusion detection.

### 8.5 Lack of explainability-oriented annotation

Explainability has emerged as an important factor especially in safety-sensitive IIoT and CPS systems due to the increased use of complex deep learning architectures in IDS solutions. But the current datasets seldom have such annotations that can be used to inform explainable intrusion detection, which can be attack intent, causal or process level impact.

Consequence:

It is worth noting that the IDS decision is still not completely understandable to the operators and stakeholders, which lowers confidence in the implemented solution, hinders the use of the forensic analysis, and restricts the use of the adopted solution in industrial settings, where interpretability is among the regulatory demands.

### 8.6 Over-reliance on single-dataset evaluation

The typical approach to IDS research is to apply the detection model to a single dataset that is often paired with a long process of hyperparameter tuning. This is an implicit assumption of the representativeness of the datasets, and disregards cross-dataset variability.

Consequence:

As a result, the IDS models are closely linked with the specific characteristics of the datasets, which means that they cannot be transferred and their robustness is low when they are introduced to other working conditions.

### 8.7 Inadequate standardized protocols for dataset evaluation

There is no consensus on the protocols to be used for IDS datasets, particularly regarding the data splitting strategy, imbalance handling, and metric selection. Therefore, performance metrics from different studies are usually not comparable.

Consequence:

The lack of standardized evaluation practices undermines reproducibility and makes it difficult to objectively assess the progress of IDS research.

## 9 Future research directions for IoT and IIoT IDS datasets

These limitations in existing IoT and IIoT intrusion detection datasets imply that future progress is unlikely to depend on incremental model improvements but rather on fundamentally revisiting the ways in which datasets are designed. Key research directions toward enabling realistic, scalable, and deployment-ready IDS evaluation in next-generation environments include the following:

### 9.1 Digital twin-driven dataset generation

Future intrusion detection datasets should be created by leveraging digital twin technologies to emulate complex environments, such as IoT and IIoT. Digital twins allow for the synchronized modeling of cyber components and physical processes, enabling researchers to study realistic operational behavior, as well as adaptive and multi-stage attacks. Unlike static testbeds, digital twins can provide endless evolutions of system states, workloads and threat behaviors. A dataset of this kind would allow the testing of IDSs under realistic settings, considering process-aware attacks, cascading failures, and stealthy manipulations that are difficult to capture using traditional dataset generation approaches.

### 9.2 Edge-cloud collaborative IDS datasets

As IoT and IIoT deployments increasingly move toward edge-cloud architectures, future datasets must reflect distributed data generation and analysis pipelines. Existing centralized datasets do not model latency constraints, partial observability, or heterogeneous computational capabilities across the layers of the edge and cloud.

Next-generation datasets are expected to support hierarchical traffic representation, where raw or fine-grained data are available at the edge and aggregated or anonymized features are processed in the cloud. This would enable research related to collaborative IDS strategies, adaptive offloading, and resource-aware intrusion detection.

### 9.3 Transformer-ready sequential and long-horizon datasets

The growing interest in Transformer-based IDS models calls for datasets that preserve long-range temporal dependencies and contextual continuity. Most current datasets involve short and independent sampled flows that cannot be used with attention-based structures. Subsequent data sets must be modeled in a way that would allow them to capture session-level and cross-session time associations to teach models about changing attack patterns, rate-slack attacks, and long-term behavioral anomalies. Datasets suitable for transformer models would help significant assessment of attention mechanisms in intrusion detection.

### 9.4 Federated and privacy-preserving dataset design

Due to the growing regulatory and privacy limits, federated learning has become an appropriate direction in implementing IDS. Nevertheless, lack of federated datasets is one of the biggest challenges to the development of this field.

The next-generation data sets ought to be distributed (partially or entirely) across devices, places, or organizational boundaries, therefore, making it possible to conduct a realistic analysis of federated IDS models when non-independent and non-identically distributed. This kind of data would be a great contribution to the study on the preservation of privacy in intrusion detection, without unrealistic assumptions of synthetic data.

### 9.5 Explainability-oriented dataset annotation

Using IDS on safety-critical IIoT and CPS meetings needs interpretable and transparent decision-making. Thus, the next data sets should not be limited to binary or categorical identity of attacks but provide explanability-friendly annotation. These can obtain the intent of attack, components of the affected systems, causal relationships and even physical implications. Understandable datasets would aid in building reliable IDS models that would offer meaningful information to operators, auditors and regulators.

### 9.6 Continual learning and dataset evolution

Statics cannot be used as an adequate measure of the performance of IDS models in dynamically evolving environments. The datasets to be used in the future must be designed to accommodate scenarios of continual learning where new forms of attacks as well as device behaviors and protocol updates will be introduced. The ability to expand datasets incrementally and label them by time results in such datasets being able to address the challenge of IDS resiliency concept drift and changing threat environments, which are at the core of real-world IoT and IIoT security.

## 10 Conclusion

This review is a dataset-based critical examination of Internet of Things (IoT)/Internet of Things (IIoT) intrusion detection (IDS) datasets; however, because the variation in the quality, realism, and design of datasets employed to test IDSs is a fundamental limiting aspect to the research advancement of the IDS, emphasis is placed on the datasets used in testing. Although machine and deep learning have recently improved, this study demonstrates that much of the reported improvement is skewed more by the nature of the dataset than actual improvement in intrusion detection ability. This review highlights, through a methodical taxonomy and in-depth comparative analysis, that a dataset determines the outcome of the IDS by defining the level of threat, evaluation bias, and relevance of deploying that dataset. IoT botnet-centered datasets, including BoT-IoT and N-BaIoT, are ideal for benchmarking high-volume attack detection and have been repeatedly found to produce inflated accuracy owing to the high level of class imbalance and repetitiveness of attack patterns. In contrast, IIoT- and cyber-physical system (CPS)-oriented datasets, such as SWAT, WADI, Edge-IIoTSet, and ToN_IoT, are far more realistic and protocol-fidelic and thus better suited to test IDS solutions that are to be deployed in safety-critical industrial contexts, albeit with significantly lower and more realistic performance. Conventional network intrusion datasets are also useful for comparison with the baseline but require caution when applying the findings to the IoT and IIoT worlds. Among the key findings of this survey is the fact that accuracy is insufficient to imply the efficiency of an IDS. A thorough evaluation should be conducted to give the datasets due attention in terms of realism, time continuity, protocol semantics, and the distribution of classes. It further reduces the generalizability of existing IDS studies by the popular tradition of single-dataset testing and by applying complex learning models blindly. Lastly, this study illustrates the necessity to change the paradigm to the next-generation dataset design. Future data must be adaptive, temporally changing, privacy-conscious, and explainable to accommodate new IDS paradigms, such as federated learning, edge-cloud collaboration, and transformer-based sequential modeling. The research community can work toward intrusion detection solutions that will offer robust, trusting and operationally significant security to IoT and industrial IoT systems by concentrating on quality of the data sets, matching deployment to the needs of IoT systems.
